# Supplementary material for: Multispectral UV Imaging on Capacitive CMOS Arrays Enabled by Solution‐Processed Metal‐Oxide Nanoparticles
Source: Adv Sci (Weinh). 2026 Jul 31:e76880. Online ahead of print. doi: 10.1002/advs.76880 (PMC13427230; doi:10.1002/advs.76880)
Supplement: Supplementary file 1 — Supporting File: advs76880‐sup‐0001‐SuppMat.docx. [file ADVS-9999-e76880-s001.docx]

**Multispectral UV Imaging on Capacitive CMOS Arrays Enabled by Solution-Processed Metal-Oxide Nanoparticles**

*Suman Kundu^1,2^*, Tao Shen^1,2^, Kai Betlem^1,2^, Murali K Ghatkesar^2^, Peter G Steeneken^2^, Frans P Widdershoven^1,3^**

1. Department of Microelectronics, Faculty of Electrical Engineering, Mathematics and Computer Science, TU Delft, Netherlands
2. Department of Precision and Microsystems Engineering, Faculty of Mechanical Engineering, TU Delft, Netherlands
3. NXP Semiconductors, Technology & Operations/CTO office, High Tech Campus 46, Eindhoven, Netherlands

Email: [S.Kundu@tudelft.nl](mailto:S.Kundu@tudelft.nl), [frans.widdershoven@nxp.com](mailto:frans.widdershoven@nxp.com)

**Table T1.** Comparison of the performance of state-of-the-art UV imagers and detectors

| **Sl. No.** | **Reference** | **Spectrum coverage** | **Multispectral**  **UV detection** | **UV-active materials** | **CMOS-compatibility** | **Respon-sivity**  **(A W^-1^)** | **NEP**  **(×10^-15^**  **W Hz^-1/2^)** | **Imaging ability** |
| --- | --- | --- | --- | --- | --- | --- | --- | --- |
| 1 | **This work** | **240-400 nm** | **Yes,**  **Selective discrimination of UV-A, UV-B, and UV-C bands shown within a single CMOS chip** | **ZnO,**  **SnO_2_ and**  **Ga_2_O_3_ nanoparticles** | **Fully CMOS compatible** | **** 0.015- 0.13**  **mF W^-1^** | **17-138** | **Yes,**  **with 32×32 pixels**  **each in 3 arrays** |
| 2 | Adv. Mater. 2022, 34, 2109498 | 250-1100 nm | Not shown | ZnMgO downconverters | Compatibility with a Si-photodiode was shown | 0.0075  at 260 nm with a Si-photodiode | - | Not shown |
| 3 | Microsyst. Nanoeng. 2022, 8, 114 | 200-400  nm | Not shown | SiC | Yes | - | - | Yes,  with 64 pixels |
| 4 | ACS Nano 2022, 16, 11, 18822–18829 | 300 nm-  2.5 µm | Not shown in UV range | CsPbX_3_ (X = Br, Cl, I) quantum dots downconverters | Yes.  A transparent ITO top contact needs to be fabricated for UV-pixels | 0.25  at 300 nm | - | Yes,  with 320×256 pixels |
| 5 | Adv. Sci. 2021, 8, 2101106 | 200-265 nm | Not shown | a-Ga_2_O_3_ | Not shown | 733 | 3.5 | Yes,  with 32×32 pixels |
| 6 | Adv. Mater. Interfaces 2024, 12, 2300371 | *220-400 nm | Not shown | GaN/Ga_2_O_3_  heterojunctions | Not shown | 0.1 | - | Yes, with 64 pixels |
| 7 | Adv. Photonics Res. 2022, 3, 2200192 | 254 nm | Not shown | ε-Ga_2_O_3_ | Not shown | 286.2 | - | Yes, with 20 pixels |
| 8 | PhotoniX 2024, 5, 5 | 120-220 nm | Not shown | AlN-SiC | Not shown | 0.054 | - | Yes, with 8×8 pixels |
| 9 | IEEE Sens. J. 2021, 21, 13, 14815-14821 | 232-250 nm | Not shown | Ga_2_O_3_ | Yes | 51 | - | Not shown |
| 10 | Adv. Mater. 2022, 34, 2106923 | *200-280 nm | Not shown | a-GaO_X_ | Not shown | 66.7 | - | Yes, with 10×10 pixels |
| 11 | Sens. Actuators A Phys. 2018, 269, 363–368 | 300-400 nm | Not shown | MgZnO nanorods | Not shown | 5.91 × 10^−4^ | 335,000 × 10^−15^  W | Not shown |
| 12 | ACS Photonics 2025, 12, 7, 3653–3661 | 200-300 nm | Not shown | β-Ga_2_O_3_/LaAlO_3_/Nb:SrTiO_3_ heterojunction | Not shown | 853.6 | 0.7 | Not shown |
| 13 | Appl. Phys. Lett. 2008, 92, 101120 | 200-450 nm | Not shown | GaN | Not shown | 0.103 | 33 | Not shown |
| 14 | IEEE Trans. Electron Devices 2023, 70, 7, 3468-3474 | *200-280 nm | Not shown | AlGaN | Not shown | 3.42 | 127 × 10^−15^ W | Not shown |
| 15 | Adv. Optical Mater. 2014, 2, 348–353 | 300-700 nm | Not shown | C-TPD:ZnO | Not shown | 1.28 | 34 | Not shown |
| 16 | Adv. Funct. Mater. 2022, 32, 2206993 | *250-400 nm | Not shown | TCTA/fullerene | Not shown | 572 | *730 | Not shown |
| 17 | Sci. Rep. 2021, 11, 10859 | *300-700 nm | Not shown | GaN | Not shown | 35.4 | 100 | Not shown |
| 18 | Nanotechnology 2022, 33, 305201 | 300–950 nm | Not shown | MoSe_2_/p-GaN | Not shown | 130 | 18 | Not shown |
| 19 | Small Methods 2024, 8, 2301767 | 325 nm | Not shown | (PMA)_2_PbCl_4_ | Not shown | 0.247 | 1,430 | Not shown |
| 20 | IMX487-AAMJ-C, Sony  (Commercial UV imager) *** | 200 – 400 nm | Not shown | - | Yes | - | - | Yes,  with 2840 × 2840 pixels |
| 21 | S16101, Hamamatsu  (Commercial UV imager) *** | 245 - 1100 nm | Not shown | - | Yes | - | - | Yes,  with 1280 × 1024 pixels |
| 22 | AS7331, ams-OSRAM  (Commercial spectral UVA/B/C sensor) *** | 230 - 430 nm * | Yes,  Three channels at 260, 300 and 360 nm | - | Not shown | - | - | No |

*Estimated values, ** The responsivity unit (F W^-1^) of the present capacitive sensor is different from the conventional charge-based photodetectors (A W^-1^) ***Commercial UV imaging systems are typically designed for broadband detection across the 200–400 nm spectral range and emphasize high frame rates and high spatial resolution using UV-enhanced CMOS image sensors. Examples include sensors such as the Sony IMX487 series and Hamamatsu S16101. In contrast, multispectral imaging within the UV band has been explored to a much lesser extent. While some commercial detectors can selectively detect UV-A, UV-B, or UV-C radiation (e.g., AS7331 spectral UV sensor), these devices typically operate as point detectors without spatial imaging capability. In the present work, we demonstrate both band-selective detection and spatial imaging across the UV-A, UV-B, and UV-C spectral regions using a CMOS capacitive sensor array that is post-functionalized with different metal-oxide nanoparticles. This late-stage functionalization approach enables tuning of the spectral response through material selection and provides a flexible platform for multispectral UV imaging without modifying the underlying CMOS electronics.

**
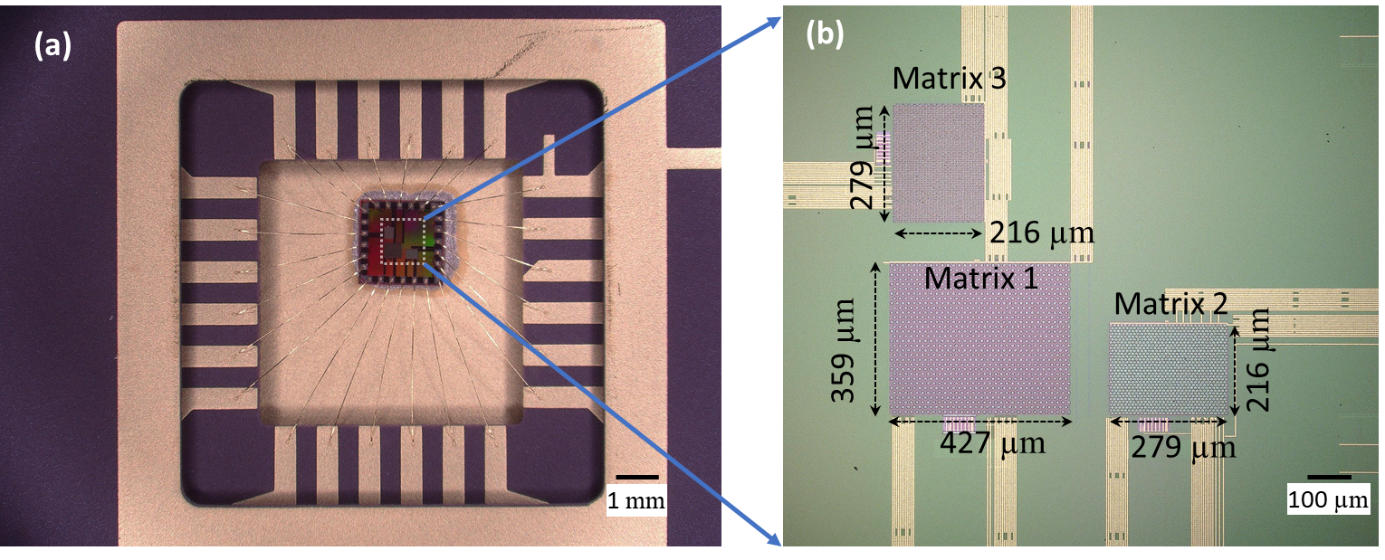
**


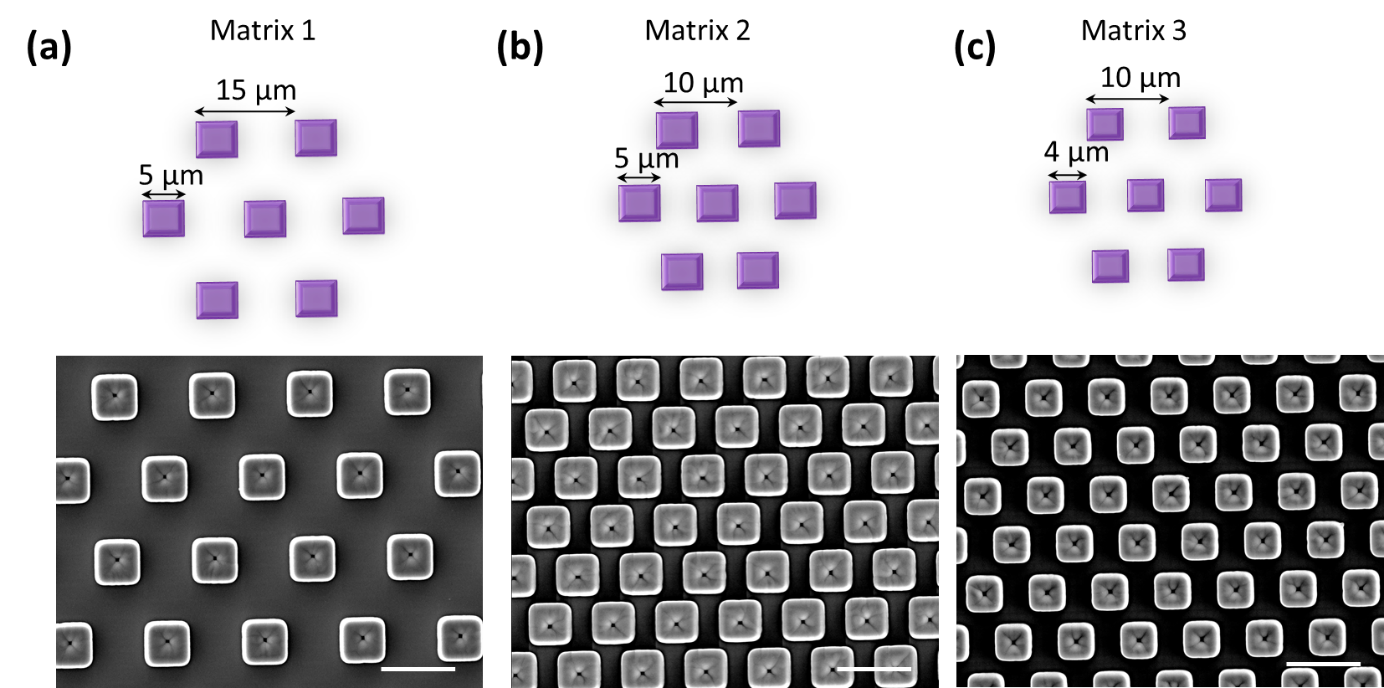
**Figure S1.** (a) Image of the CMOS chip containing the PCS arrays. (b) Optical microscopy image of the three matrices with their dimensions. The fabricated chip has overall dimensions of ~ 1.5×1.5 mm^2^, within which Matrices 1, 2, and 3 occupy areas of 427×359 µm^2^, 279×216 µm^2^, and 279×216 µm^2^, respectively.

**Figure S2**. The schematic representation of the dimensions of the electrodes and spacing between them in the (a) Matrix 1, (b) Matrix 2 and (c) Matrix 3. Corresponding Field Emission Scanning Electron Microscopy (FESEM) images of the sensor pixels of each matrix (1, 2, and 3) are shown below. Scale bars in a, b, and c represent 10 µm.


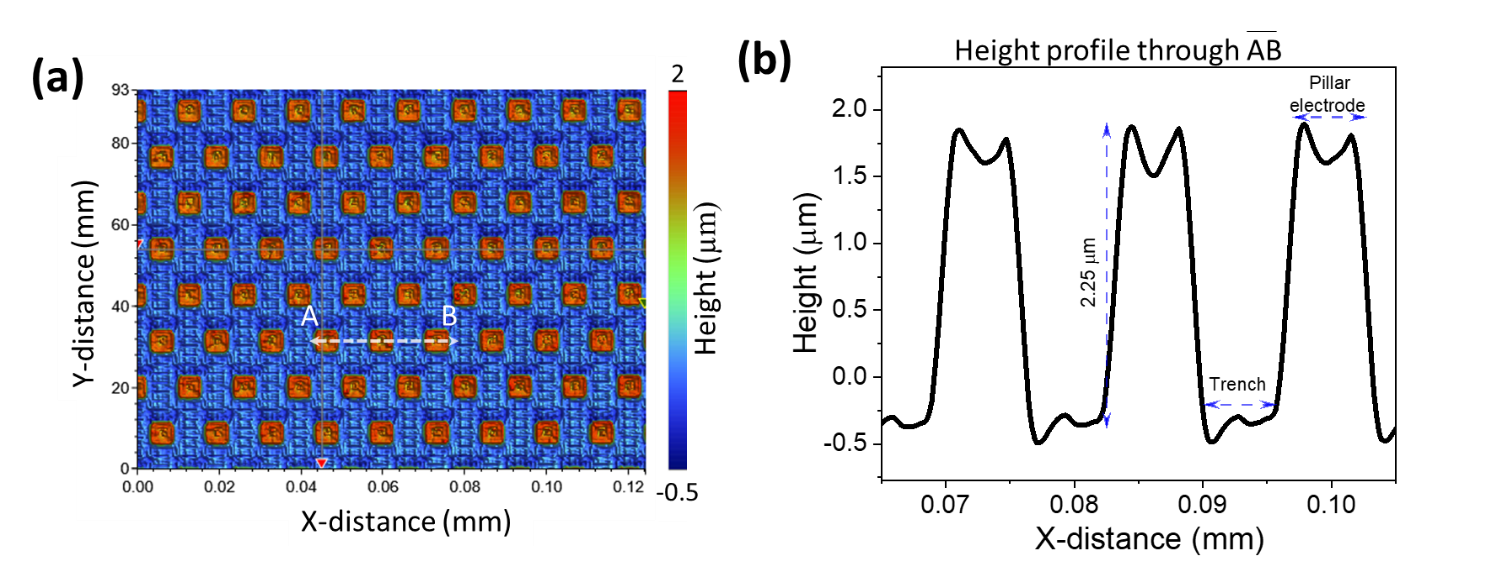
**Figure S3**. (a) Optical height-profilometry of a portion of sensor pixels in Matrix 1. (b) The height profile through the line AB in (a), containing three electrode pillars and trenches in-between. The depth of the trenches from the top of the pillar is measured to be around 2.25 µm, as indicated inside.

**Note 1. Working principle of the PCS arrays**

The equivalent electrical circuit of a sense electrode of PCS array is shown in Figure S4. The sense node N is connected to the sense electrode. 𝐶_𝐶_ represents the coupling capacitor through the protective layer over this sense electrode. 𝐶_𝑃_ represents the parasitic capacitor. 𝐶_𝑀_ and 𝑅_𝑀_ represent the material in the passivation layer valleys, which can be either sensing materials or air (𝑅_𝑀_ = ∞ for air). Switches S_T_ and S_D_ toggle the sense voltage 𝑉_N_ between the charge transfer voltage 𝑉_𝑇_ and ground with a repetition frequency 𝑓_N_. In each cycle an amount of charge 𝑄 is transferred from the charge transfer node T to the ground.

The average charge transfer current is 𝐼_𝑇,𝑎𝑣_ = 𝑓_N_𝑄

For 𝑓_N_ ≫ 1⁄(2𝜋𝑅_𝑀_𝐶_𝑀_), where the current through 𝑅_𝑀_ can be neglected, the transferred charge per charge/discharge cycle is 𝑄 = 𝑉_𝑇_ (𝐶_𝑅_ + 𝐶_𝑃_), where 𝐶_𝑅_ = 𝐶_𝐶_𝐶_𝑀_ /(𝐶_𝐶_ + 𝐶_𝑀_) is the read path capacitance (𝐶_𝑀_ in series with 𝐶_𝐶_).

Electrodes are functionalized by filling their surrounding channels with materials that change their dielectric properties when exposed to external stimuli like UV light. In the following we call these materials ‘sensing inks’. To suppress drift and low-frequency 1/f noise in the read-out circuits we apply correlated double sampling (CDS) by subtracting the average charging current of non-functionalized reference electrodes from that of functionalized sense electrodes. A change ΔI_measured_ in this CDS-corrected current, caused by exposure to UV light, is related to a change ΔC in the sense capacitance: ΔI_measured_ = 𝑓_N_ × ΔC × V_T_. We solve ΔC from this equation. The frequency 𝑓_N_ was kept at 40 MHz and V_T_ = 0.9 V.

**
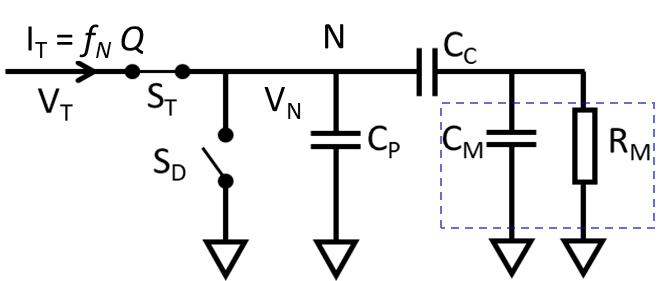
**The circuit uses the in-pixel switching electronics shown in Figure S5. Readout is performed through a cascaded PMOS–NMOS current mirror configuration, each with a mirror ratio of 1:10. The output current is thus amplified by a factor of 100, converted into a voltage by off-chip transimpedance amplifiers, and digitized with 12-bit ADCs (see Figure S6). Each of the 1024 sensor pixels across the three matrices is switched sequentially (see Figure S7), producing a complete data packet of 3,072 pixels per acquisition cycle which completes with a typical sampling rate of 4.55 Hz per frame (i.e. reading all 3×1024 electrodes).


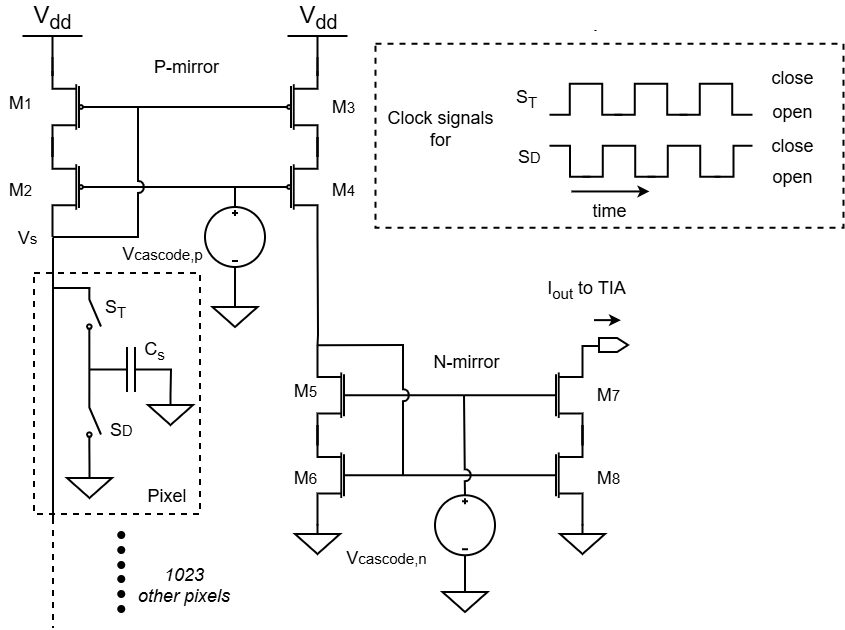
**Figure S4.** The representative image of the equivalent circuit of a single capacitive sensor pixel in PCS array. The components (R_M_, C_M_) arising due to the deposited-nanoparticles on the trenches are marked inside a blue-dashed rectangle.

**Figure S5**. The read-out circuit for the capacitive sensor pixels. The non-overlapping clock signals (dashed box) control the switches S_T_ and S_D_ for alternately opening and closing. In each matrix, 1 pixel is selected for sensing while the other 1,023 pixels are grounded. The average sense currents of the 3 selected pixels are multiplied by 100 by 3 on-chip chains of a 10x PMOS current mirror and a 10x NMOS current mirror, converted into voltages by 3 off-chip Transimpedance Amplifiers (TIAs), and digitized by 3 12-bit Analog to Digital Converters (ADCs) (see Figure S6). The resulting 3 digital codes are read in parallel by a microcontroller and transferred to a computer over a USB link. This process is repeated by scanning though the 1,024 pixels of the 3 arrays in parallel.


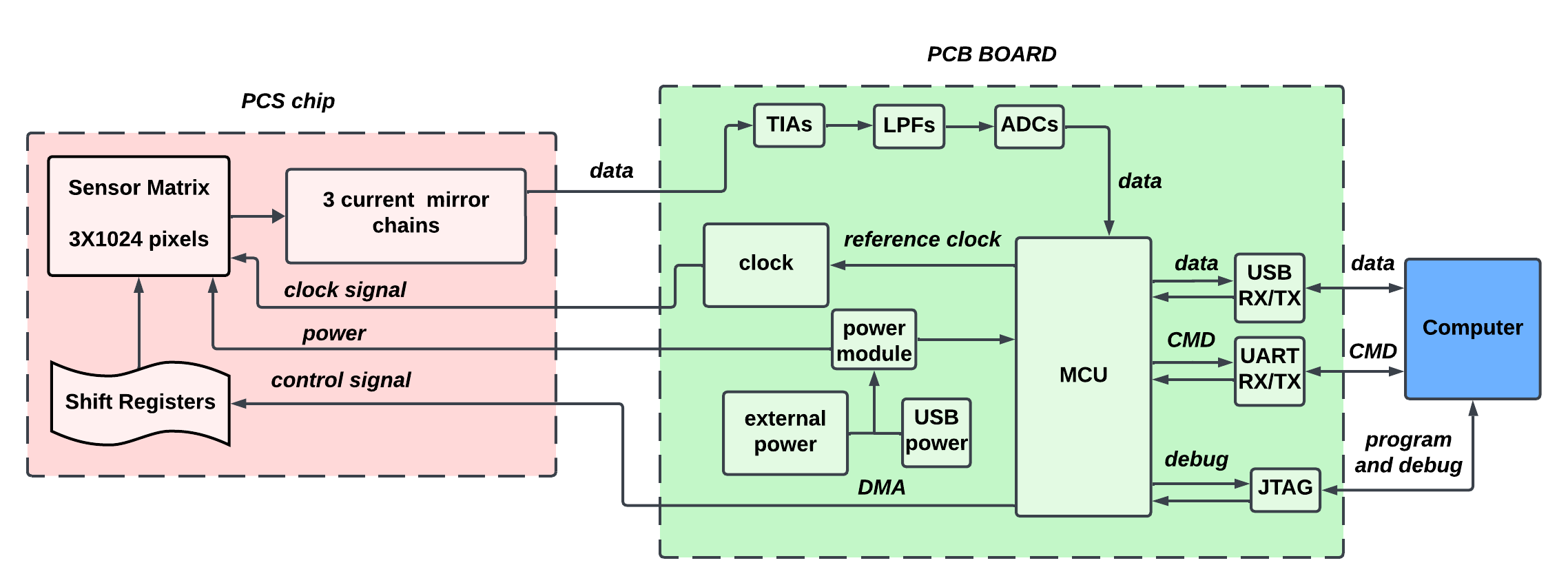


**Figure S6**. Simple block diagram of the circuits in the PCS chip and measurement board for data recording from the PCS sensor pixels. The recorded data are then analyzed in real-time using a computer.


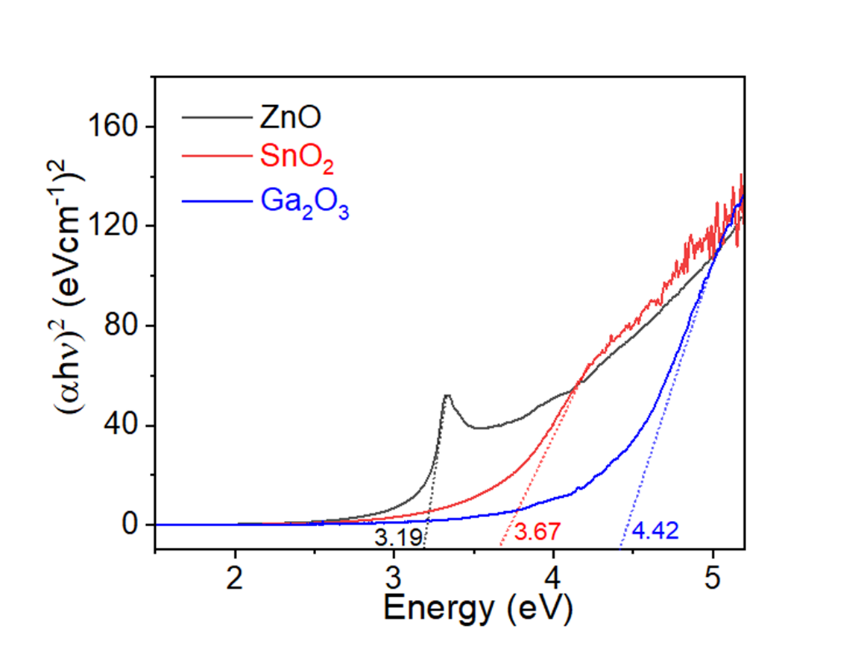

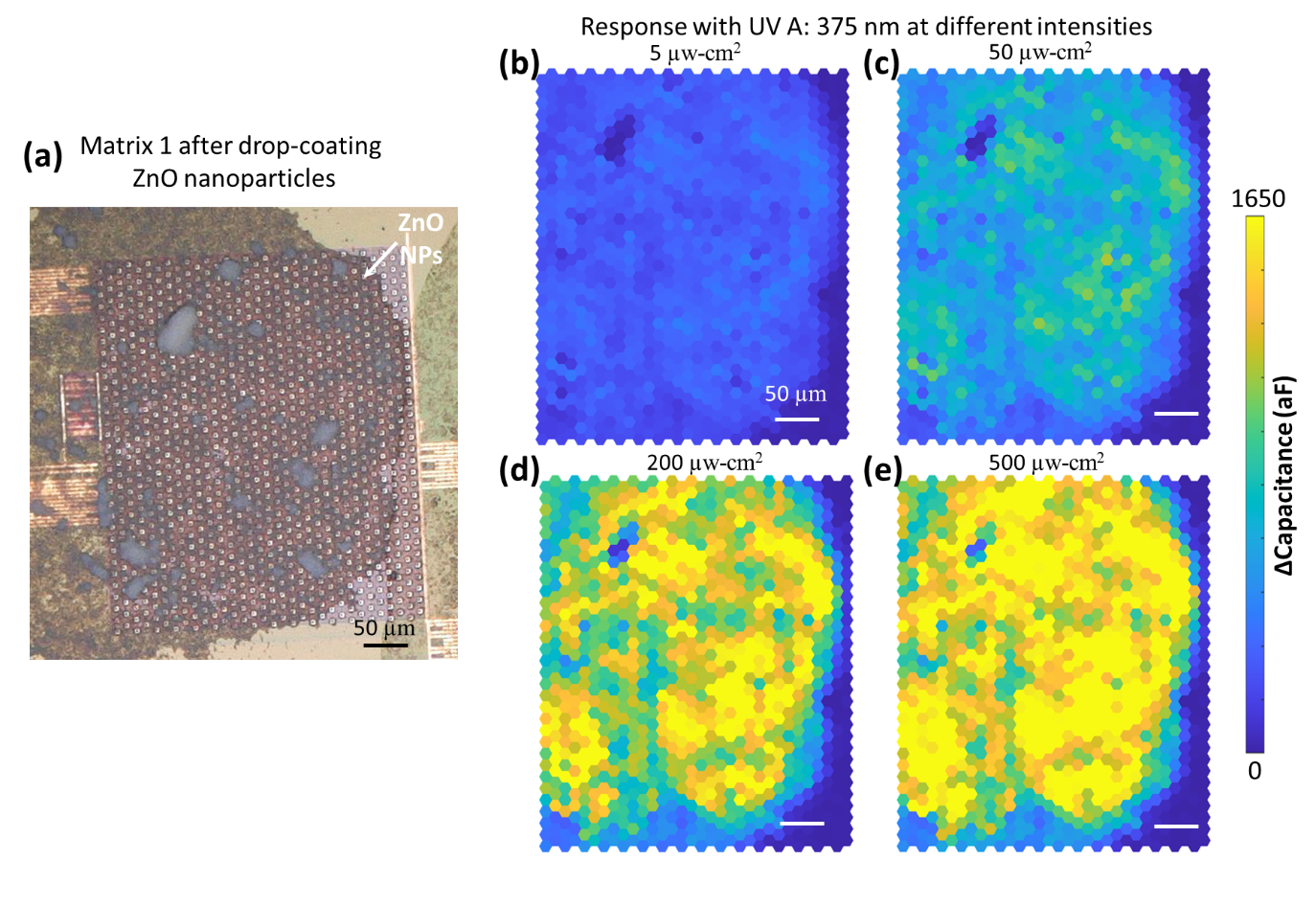
**Figure S7**. Tauc plots of metal-oxide nanoparticles (MOX NPs) used in this study. The estimated bandgap energies are 3.19 eV for ZnO, 3.67 eV for SnO_2_, and 4.42 eV for Ga_2_O_3_, as indicated inside the figure.

**Figure S8**. (a) Optical microscopy image of the PCS Matrix 1 after drop-coating ZnO NPs. (b-e) Response of the sensor pixels under UV A:375 nm exposure at different intensities as indicated on top of each figure. Scale bars in c, d, and e represent 50 µm. Capacitance scale bar for b-e is shown at right.

**
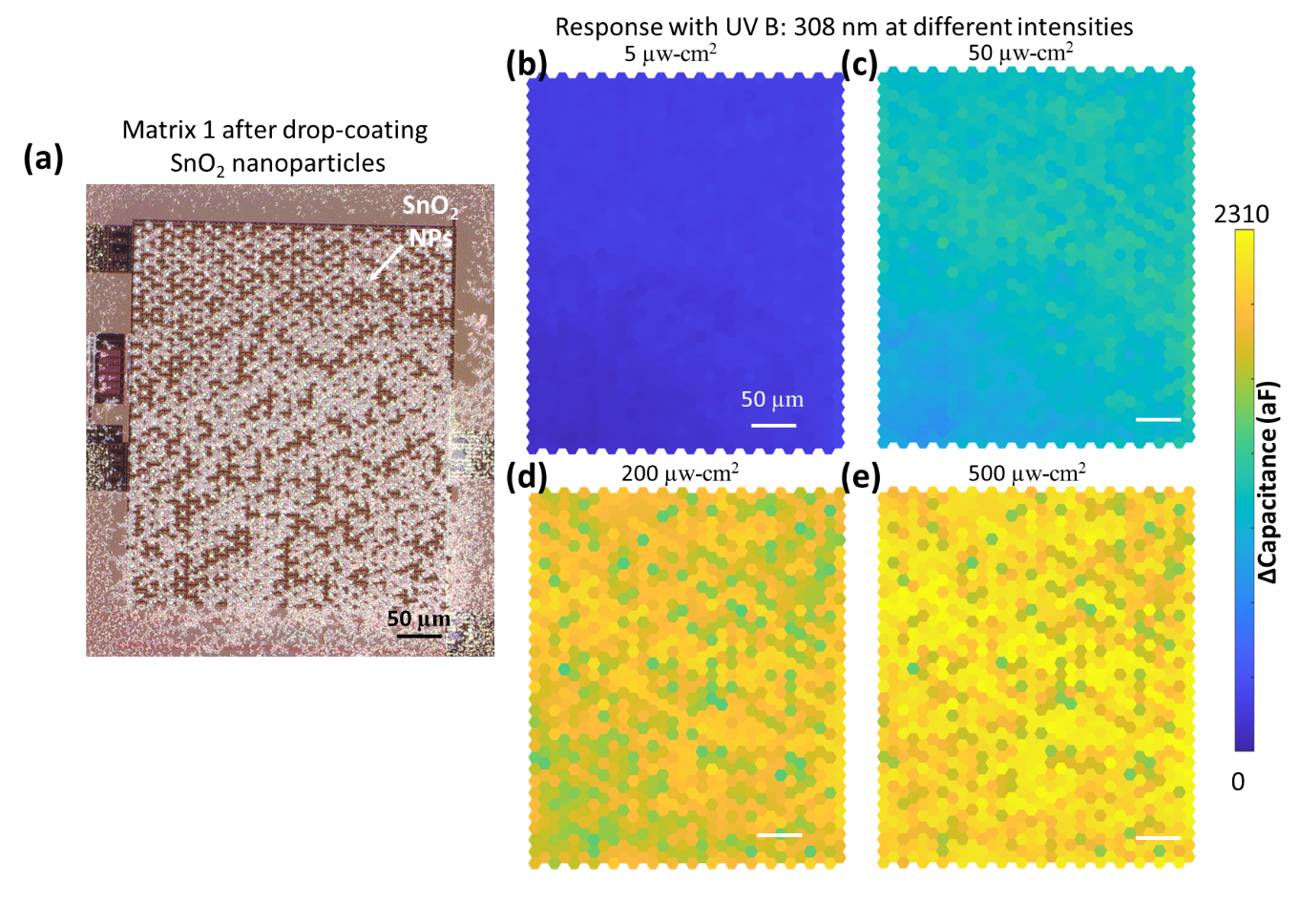
**

**Figure S9**. (a) Optical microscopy image of the PCS Matrix 1 after drop-coating SnO_2_ NPs. (b-e) Response of the sensor pixels under UV B:308 nm exposure at different intensities as indicated on top of each figure. Scale bars in c, d, and e represent 50 µm. Capacitance scale bar for b-e is shown at right.

**
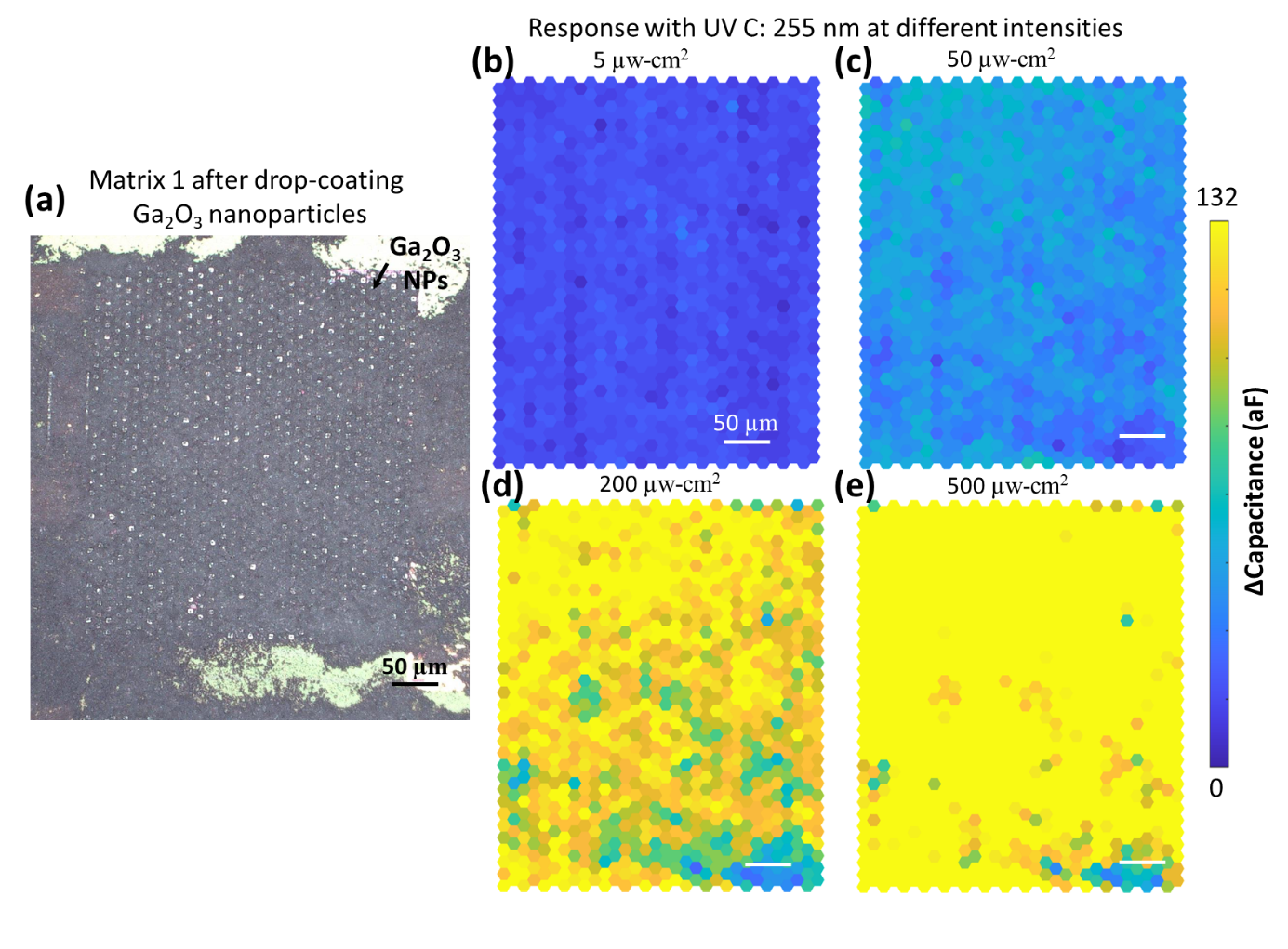
**

**Figure S10**. (a) Optical microscopy image of the PCS Matrix 1 after drop-coating Ga_2_O_3_ NPs. (b-e) Response of the sensor pixels under UV C: 255 nm exposure at different intensities as indicated on top of each figure. Scale bars in c, d, and e represent 50 µm. Capacitance scale bar for b-e is shown at right.

**Note 2.** **Differential UV-bands detection by dropcasting MOX NPs in a single chip**

To evaluate simultaneous performance from the three NP dispersions, we carefully drop-coat them onto the different regions of the PCS arrays of the same chip (Figure S11 a,b). Placing these three NP types together in a single chip facilitates differential UV band detection in a single platform. Matrix 1 was coated with SnO_2_ and Ga_2_O_3_ nanoparticles at different places, whereas Matrix 3 was partly coated with ZnO. Certain regions of the PCS array were intentionally left uncoated to serve as reference pixels. NP deposition increased the capacitance of coated pixels, as the replacement of air in the channels with higher-ε_r_ materials raised the effective dielectric constant as shown in Figure S11 c,d. We observed the highest electrode capacitance changes of ~865, 713, and 317 aF in the ZnO, SnO_2_, and Ga_2_O_3_ coated pixels, respectively. In contrast, the uncoated pixels exhibited no measurable capacitance variation, confirming the highly localized sensing capability of the PCS technology. Drop-casting produced a higher NP concentration at the periphery of each drop, with decreasing density toward the center (Figure S11 a,b), due to the coffee-ring effect. The magnitude of the capacitance change was also proportional to the NP amount (Figure S11 c,d).

The chip was then exposed to UV light emitting diodes of wavelengths 375, 308, and 255 nm, representing the UV-A, UV-B, and UV-C regions, respectively (Figure S12), as well as to visible light at 520 nm, each kept at a constant intensity of 0.25 mW cm^-2^. No response was observed under visible light (Figure 12a,b). Under UV-A illumination, ZnO coated pixels in Matrix 3 exhibited the largest capacitance increase, which scaled with amount of deposited NP, reaching a maximum of 165 aF (Figure S12d). In comparison, SnO_2_ pixels showed only a minor increase of 10-20 aF, while Ga_2_O_3_ pixels remained unresponsive (Figure S12c) with UV-A. Under UV-B, SnO_2_ coated pixels demonstrated a pronounced response, with capacitance increased up to 436 aF (Figure S12e), whereas ZnO pixels also responded but more modestly (112 aF, Figure S12f); Ga_2_O_3_ again showed no variation. Finally, under UV-C illumination, Ga_2_O_3_ coated pixels exhibited a measurable response with a maximum increase of 40 aF (Figure S12g), though this was significantly smaller than that of ZnO (100 aF, Figure S12h) or SnO_2_ (376 aF, Figure S12g).


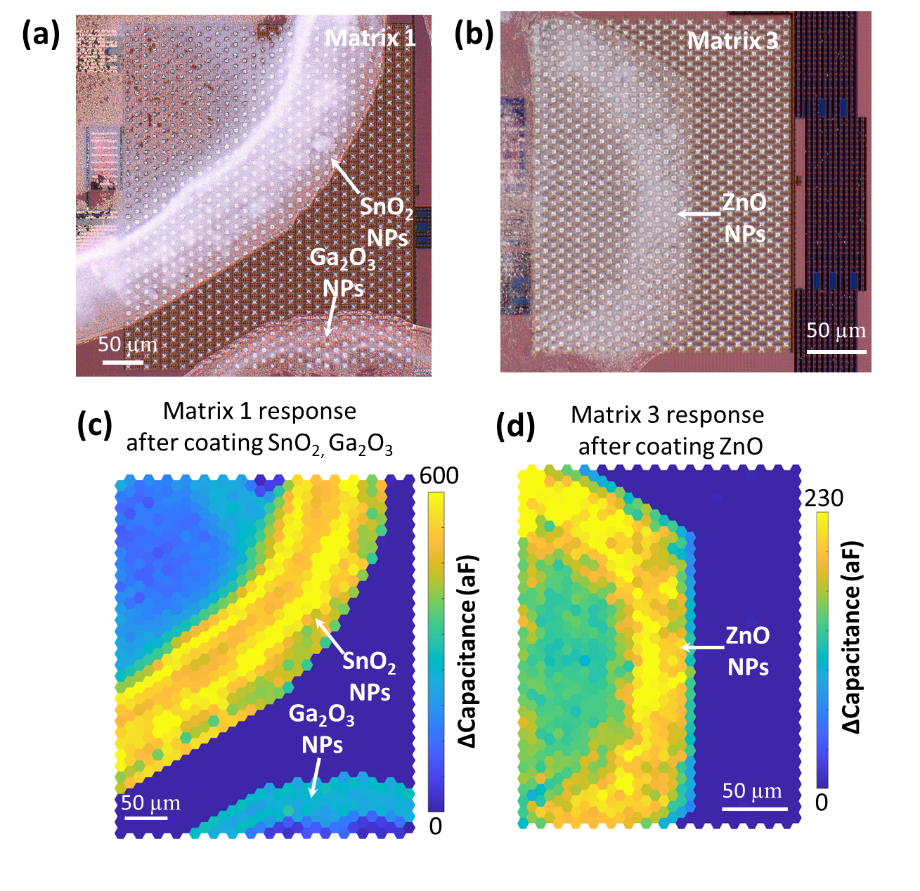
**Figure S11**. **Capacitive sensor arrays functionalized by dropcasting.** (a,b) Optical microscopy images of the device with the ZnO, SnO_2_ and Ga_2_O_3_ NPs dropcasted onto the Matrices 1, and 3. (c,d) Corresponding 2D maps of capacitance change in PCS Matrices 1 and 3 after coating the channels with SnO_2_, Ga_2_O_3_, and ZnO NPs, compared to the uncoated sensor pixels. The measurements were performed under dark conditions.


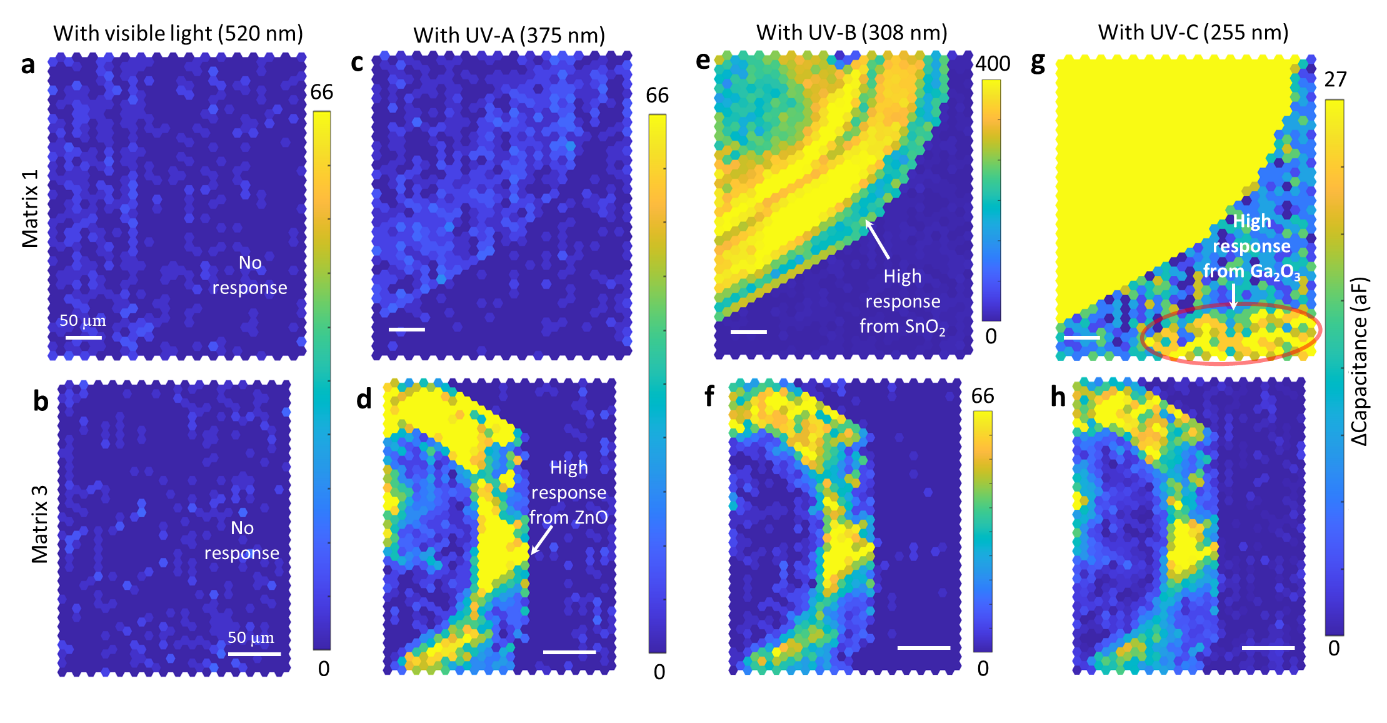
**Figure S12**. 2D capacitance-change map of the matrices 1, 3 exposed under (a, b) visible light: 520 nm, (c, d) UV-A: 375 nm, (e, f) UV-B: 308 nm, (g,h) UV-C: 255 nm, respectively, each kept at a constant intensity of 0.25 mW cm^-2^. The scalebars for respective capacitance change are shown at right. Sensor pixels coated with ZnO NPs show higher response with UV-A; SnO_2_ NPs show higher response with UV-B; Ga_2_O_3_ NPs show response only with UV-C, as indicated inside the figures. None of the MOX NPs showed response with visible light. Scale bars in c-h represent 50 µm.

**
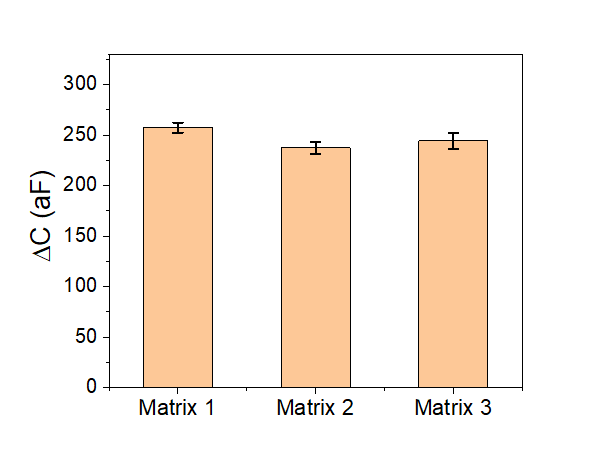
**

**Figure S13**. The optimal response from the Matrices 1, 2, and 3, observed with drop-coated Ga_2_O_3_ nanoparticles under UV-C (500 µW cm^-2^).

**
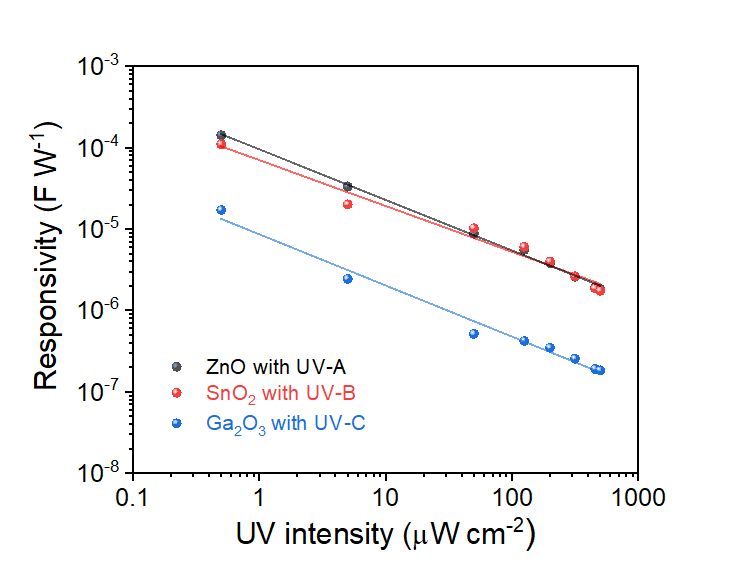
**

**Figure S14**. The optimal responsivity values obtained from ZnO (Figure S8), SnO_2_ (Figure S9) and Ga_2_O_3_ (Figure S10) drop-coated chips under UV A, B and C exposures, respectively at varying intensities (0.5-500 µW cm^-2^).


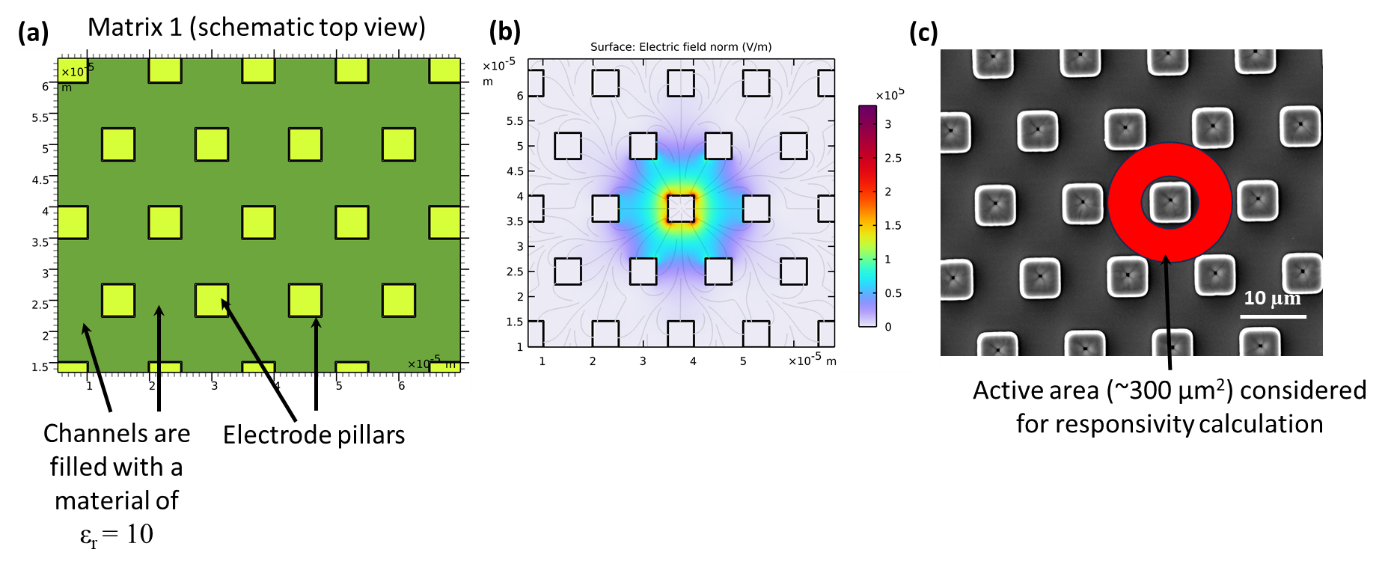


**Figure S15**. Schematic top view of the sensor pixels in Matrix 1, with the channels filled with a material of relative permittivity ε_r_ = 10, which is close to the relative permittivity of the MOX NPs used in this work. (b) Simulated electric field distribution (top view) of the sensor pixels using COMSOL Multiphysics. Only the centre electrode is positively biased, while all surrounding electrodes are grounded. (c) FESEM image of the Matrix 1. The red-marked region in c was selected for calculating the responsivity values, and it closely corresponds to the approximate active area (~300 µm^2^) with the highest electric field distribution as shown in b.


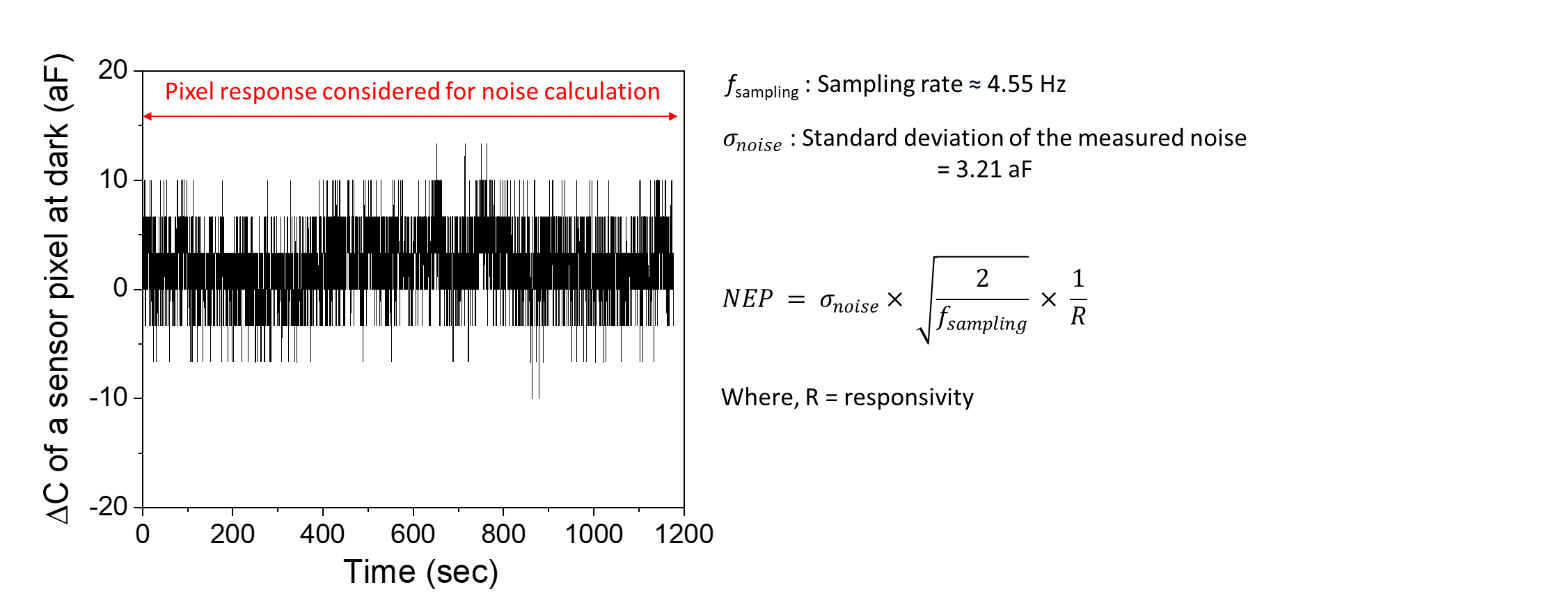


**Figure S16**. Response of a sensor pixel in Matrix 1 kept at dark condition. The standard deviation of the noise at a 4.55 Hz sampling rate was estimated to be 3.21 aF. The formula used for calculating the corresponding noise-equivalent power (NEP) is shown on the right side of the figure.


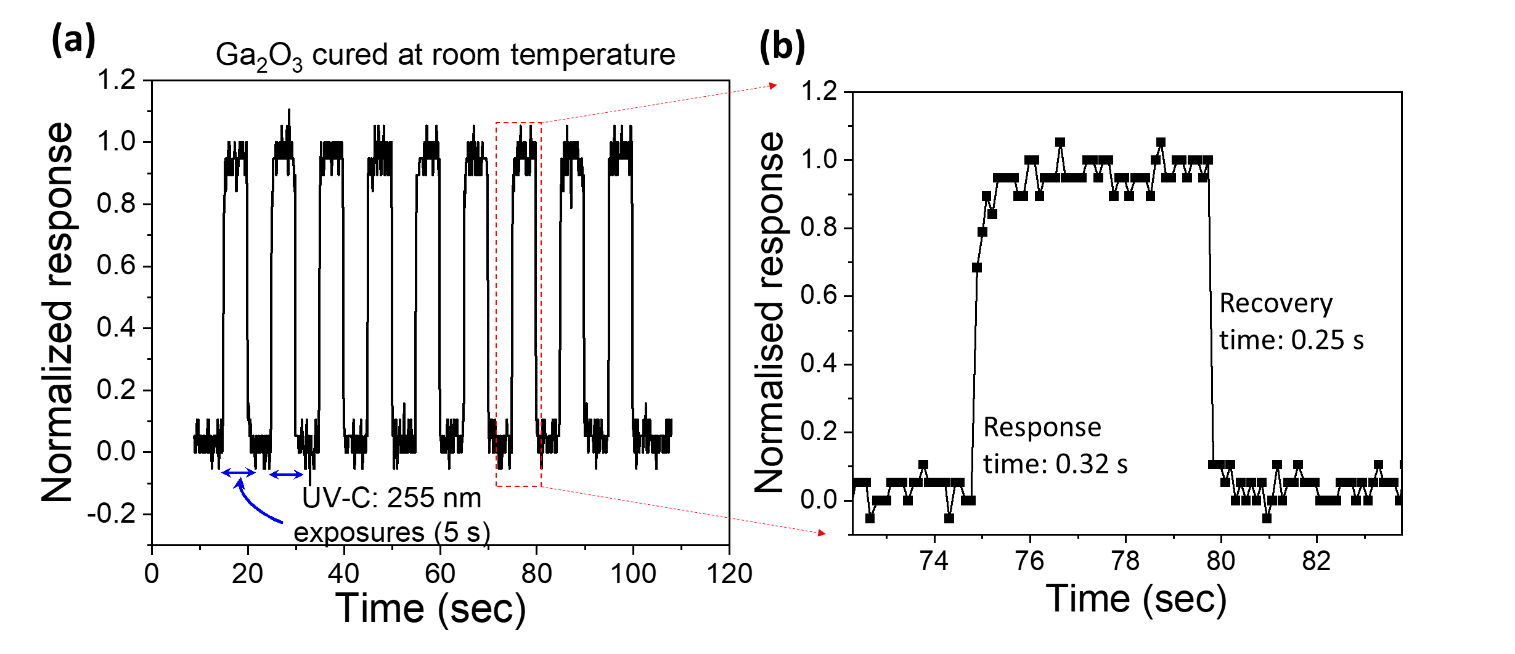

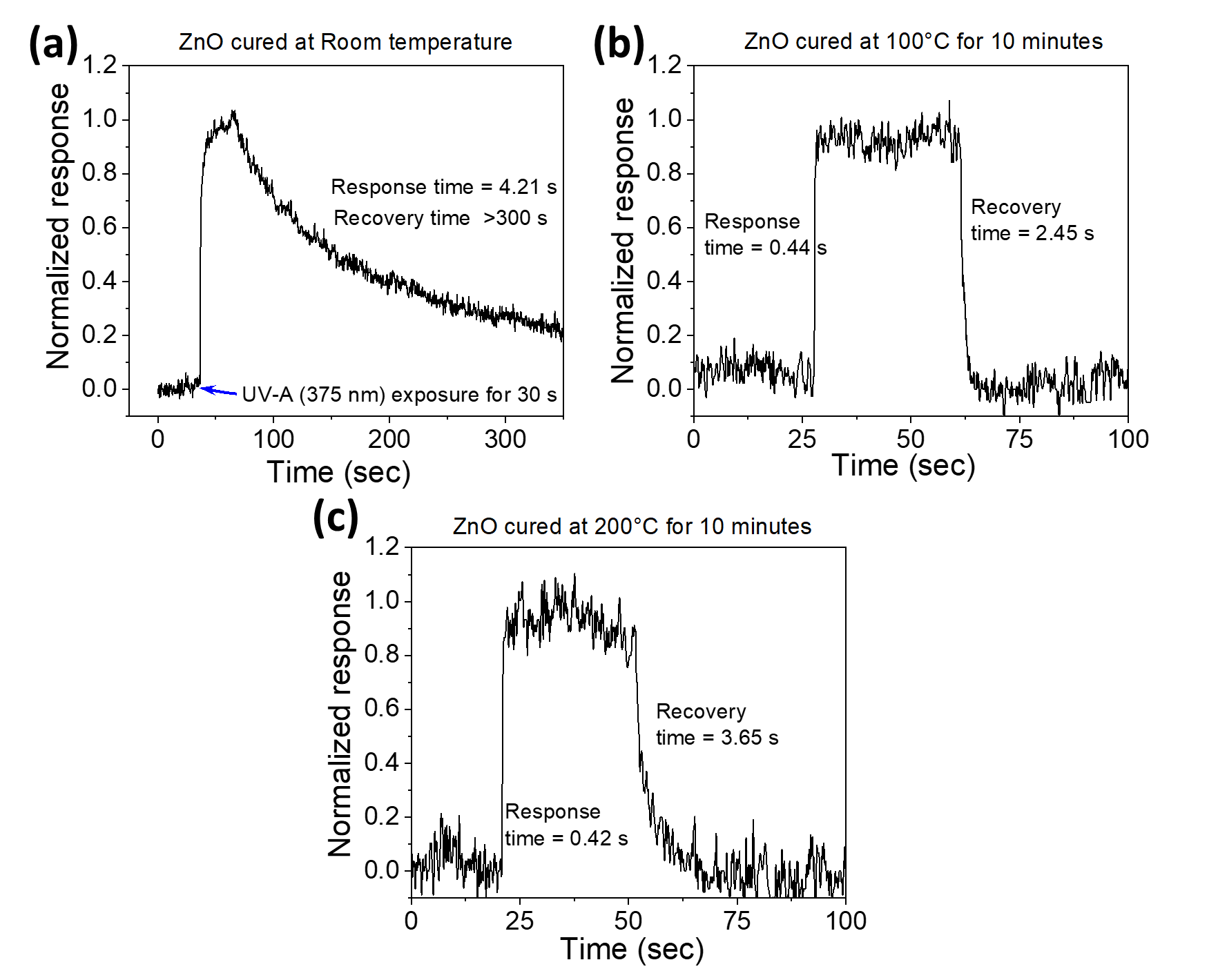
**Figure S17.** (a) Normalized responses of Ga_2_O_3_-coated sensor pixels cured at room temperature under repeated 5 s exposure cycles of 255 nm UV light at an intensity of 250 µW cm^-2^. (b) Zoomed-in view of a single exposure cycle from (a), with the corresponding response and recovery times indicated inside.

**Figure S18**. (a-c) Normalized responses of ZnO-coated sensor pixels cured at different temperatures (as indicated above each figure) under 30 s exposure to 375 nm UV light at an intensity of 250 µW cm^-2^. The corresponding response and recovery times are indicated within each plot.

**
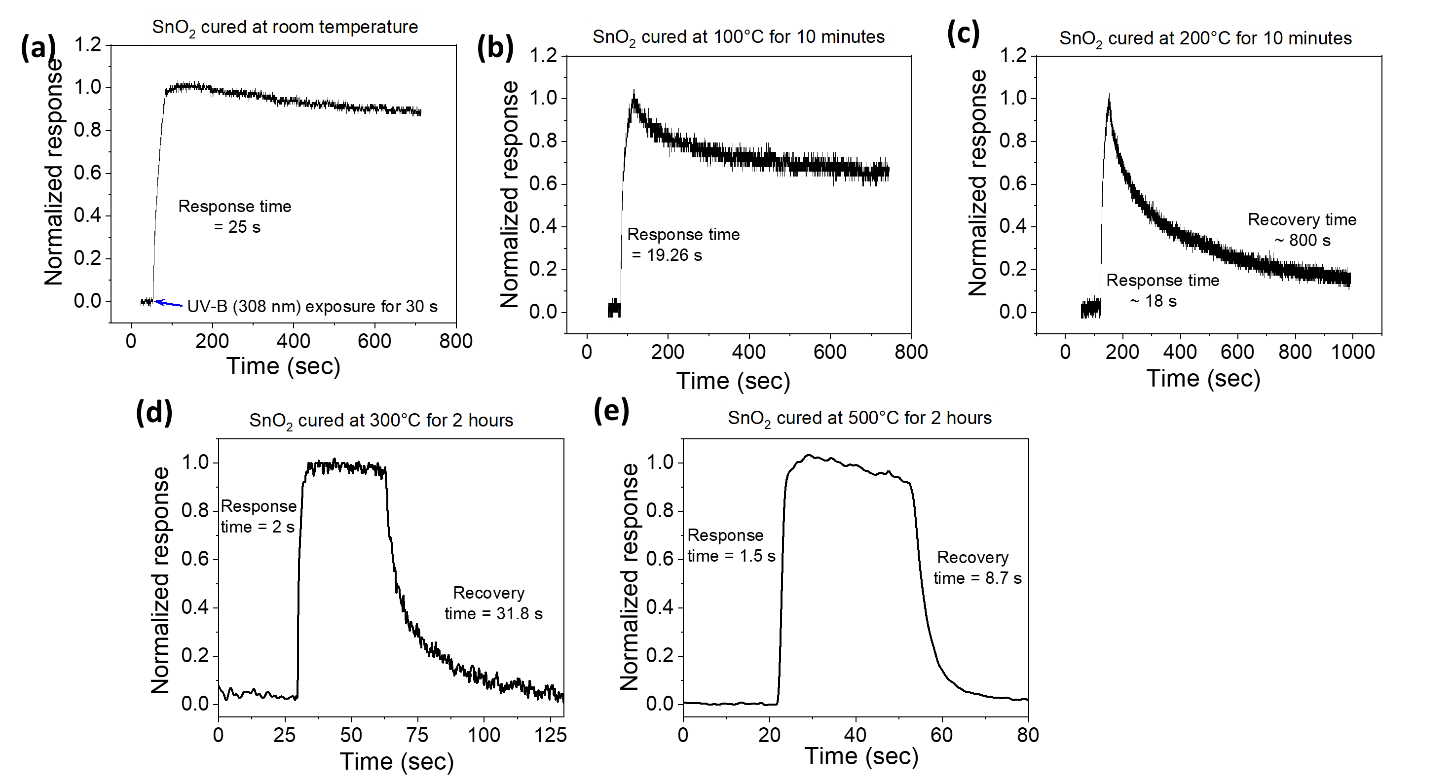
**

**Figure S19.** (a-e) Normalized responses of SnO_2_-coated sensor pixels cured at different temperatures (as indicated above each figure) under 30 s exposure to 308 nm UV light at an intensity of 250 µW-cm^-2^. The corresponding response and recovery times are indicated within each plot. For temperature upto 200 °C, the SnO_2_-coated chip was cured on a hotplate. For higher temperatures, SnO_2_ nanopowder was thermally cured separately in an oven at 300 °C and 500 °C for 2 hours in ambient air, after which the cured nanoparticles were deposited onto the sensor pixels and UV detection measurements were performed thereafter.

**Note 3.** **Simulations of capacitance change with varying relative permittivity of the deposited MOX NPs**

To investigate the capacitive behavior of the PCS platform, finite element simulations were performed for using the Electrostatics Module of COMSOL Multiphysics. The simulations allowed us to predict the capacitance changes with the varying effective relative permittivity (ε_eff_) of the metal-oxide nanoparticles deposited in the trenches.

A stationary study of electrostatics physics was applied in the simulations using the following relations:

$$\nabla\cdot D=\rho_{V}$$

$$E=-\nabla V$$

$$D=\varepsilon_{0}\varepsilon_{r}E$$

where the **D** is the displacement field, **E** is the electric field, V is the voltage potential, $\varepsilon_{0}$ is the vacuum permittivity, $\varepsilon_{r}$ is the relative permittivity of the material, and $\rho_{V}$ is the space charge density.

Three boundary conditions are applied: 1) the charge at the boundary is zero, 2) the surface potential of the central electrode is 0.9 V, and 3) the surface potential of the surrounding electrodes is zero.

The finite element analysis can give results of electric potential distribution and electric field intensity distribution. Thus, we can calculate the total electric energy as:

$W_{e}=\frac{1}{2}\int\varepsilon_{0}\varepsilon_{r}\left| \vec{E} \right|.\left| \vec{E} \right|dA$, where, dA is a volume integral.

Combining with the equation of energy stored inside a capacitor:

$W_{e}=\frac{1}{2}C\left( V_{1}-V_{2} \right)^{2}$, where $V_{1}=0.9 V and V_{2}=0 V$

The capacitance of the structure can be then estimated as:

$C$= $\frac{1}{\left( V_{1}-V_{2} \right)^{2}}\int\varepsilon_{0}\varepsilon_{r}\left| \vec{E} \right|.\left| \vec{E} \right|dA$

The simulation geometry consists of one central electrode surrounded by six ground electrodes arranged in a hexagonal configuration, with the trenches fully filled with a uniform nanoparticle film (Figure S20a-c). Based on this structure, the total capacitance was computed. ΔC was then calculated by deducting total capacitance of unfilled trenches from total capacitance of the trenches filled with the nanoparticle film.

In the simulations, we varied the effective relative permittivity (ε_eff_) of the deposited film from 1 to 200 (Figure S20d). The results show that capacitance increases with increasing ε_eff_, with the rate of increase gradually saturating at higher ε_eff_ values. This trend aligns with our experimental observations, where capacitance rises with increasing UV intensity (Figure S20e), indicating that UV exposure enhances the effective relative permittivity of the metal-oxide nanoparticles.

**
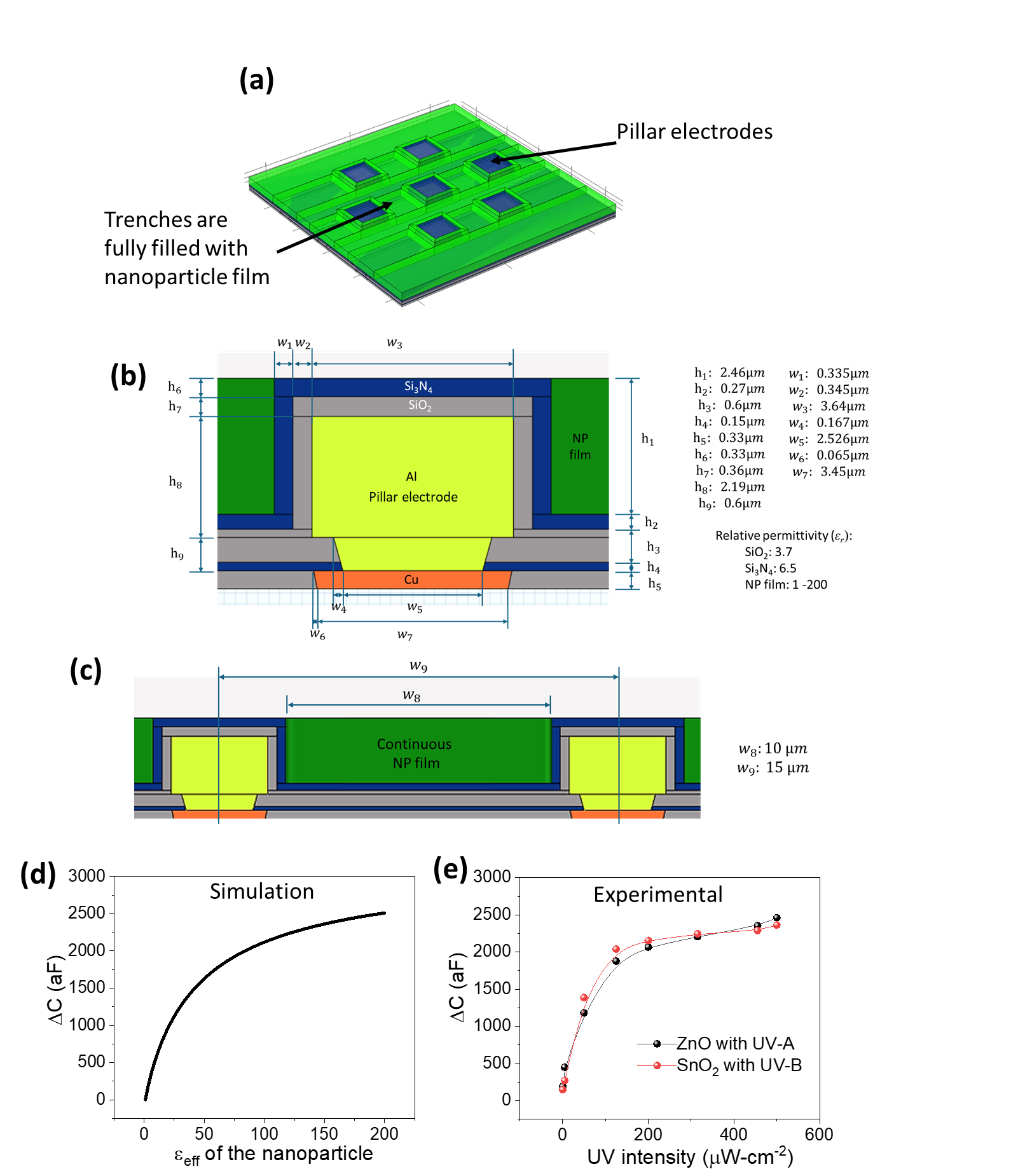
**

**Figure S20.** Simulation results: capacitance change of PCS pixels with varying relative permittivity of deposited metal-oxide nanoparticles. (a) 3D COMSOL models of sensor pixels in the PCS array. The array is fully covered with a uniform metal-oxide nanoparticle film (green-colored region). (b, c) Details of the CMOSOL model used. The values of the parameters are illustrated alongside. (d) Simulated capacitance response of pixels as a function of the effective relative permittivity (*ε*_eff_ = 1-200) of the nanoparticle film. (e) Capacitance-changes (experimentally measured) in ZnO, and SnO_2_ NPs under varying UV-A, and -B intensities, respectively.

**
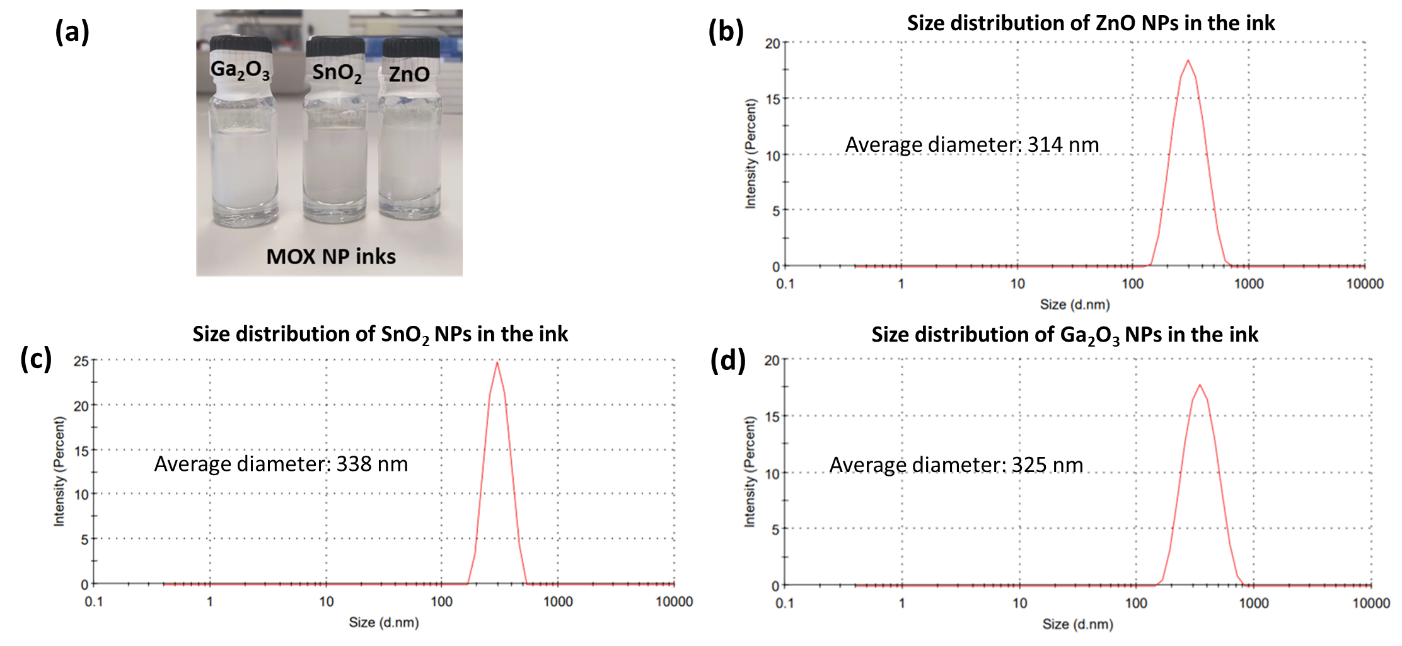
Figure S21.** (a) Image of the prepared inkjet-printable dispersions of ZnO, SnO_2_ and Ga_2_O_3_ NPs. (b-d) Dynamic light scattering (DLS) measurements of ZnO, SnO_2_, and Ga_2_O_3_ NPs in their respective inks. The average hydrodynamic particle diameters are indicated inside each plot.

**
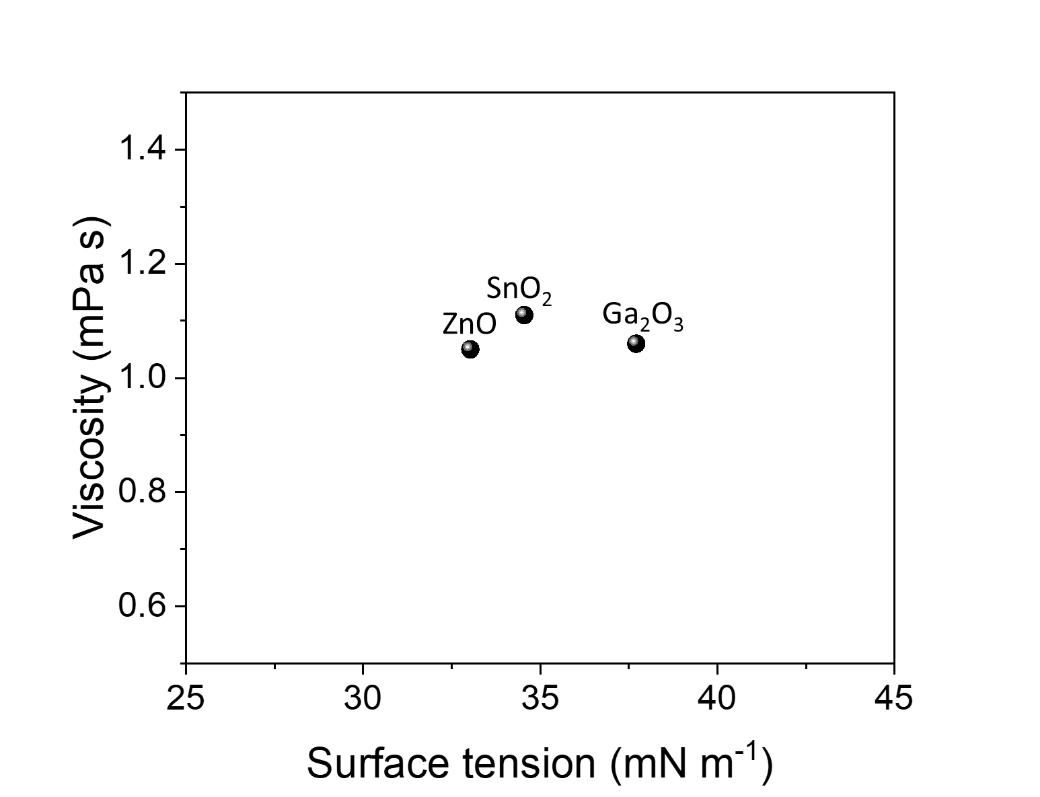
**

**Figure S22**. Viscosity and surface tension values of the prepared MOX NP inks.

**
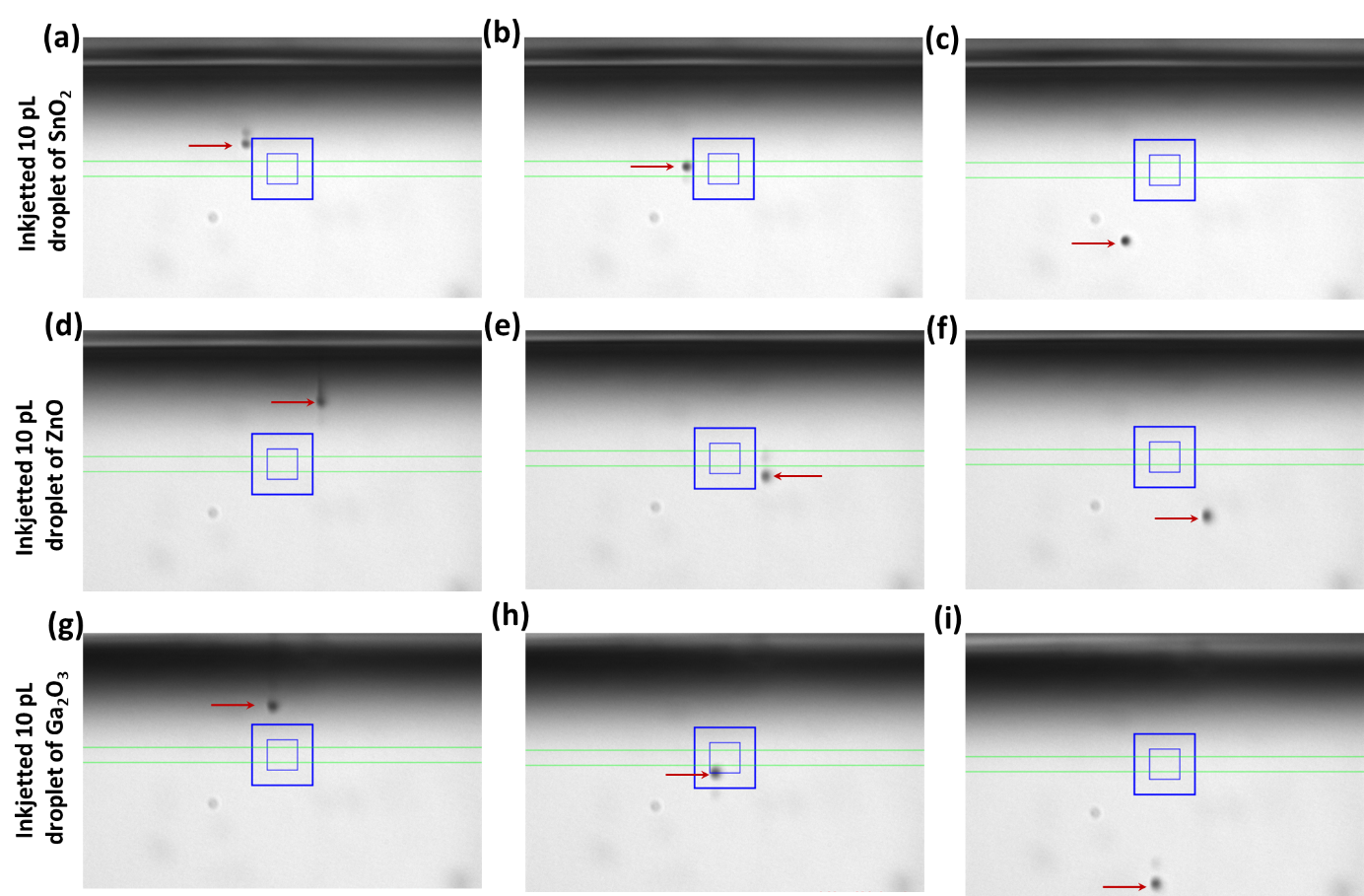
**

**Figure S23.** (a–i) High-speed stroboscopic images capturing the formation and jetting of 10 pL MOX NP ink droplets at different time intervals. The positions of the formed droplets are indicated by red arrows.


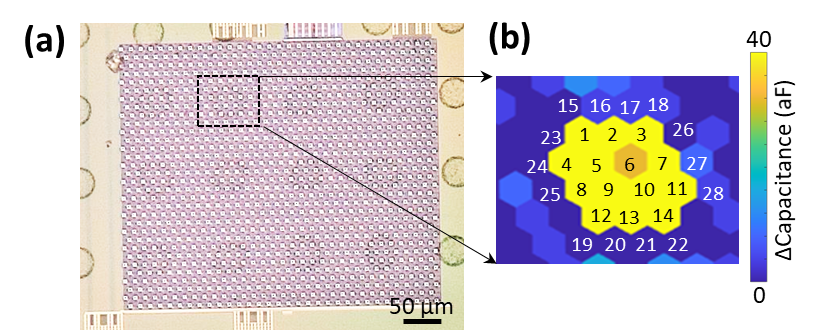


**Figure S24**. (a) Optical microscopy image of Matrix 1 inkjet-coated with SnO_2_ NP droplets (1 droplet with a volume of 10 pL) in a controlled grid. (b) Capacitive response map of rectangular region marked in a under UV-B illumination (intensity: 250 µW-cm^-2^). The responses of the individually labelled pixels (pixels 1-14 corresponding to SnO_2_-functionalized and pixels 15-28 corresponding to neighboring non-functionalized pixels) are summarized in Table T2.

**Table T2**. Capacitive response magnitudes of the pixels labelled in Figure S24

| **Pixel number**  **(functionalized)** | **Capacitance response (aF)** | **Pixel number**  **(non-functionalized)** | **Capacitance response (aF)** |
| --- | --- | --- | --- |
| 1 | 49.5 | 15 | 0 |
| 2 | 52.8 | 16 | 3.3 |
| 3 | 49.5 | 17 | 3.3 |
| 4 | 46.2 | 18 | 3.3 |
| 5 | 42.9 | 19 | 0 |
| 6 | 33 | 20 | 0 |
| 7 | 59.4 | 21 | -3.3 |
| 8 | 49.5 | 22 | -6.6 |
| 9 | 49.5 | 23 | 0 |
| 10 | 46.2 | 24 | 0 |
| 11 | 42.9 | 25 | 0 |
| 12 | 52.8 | 26 | 0 |
| 13 | 69.3 | 27 | 3.3 |
| 14 | 49.5 | 28 | 6.6 |
| Average response of functionalized pixels (aF) | 49.7 ± 7.5 | Average response of non-functionalized pixels (aF) | 0.7 ± 3.1 |

**
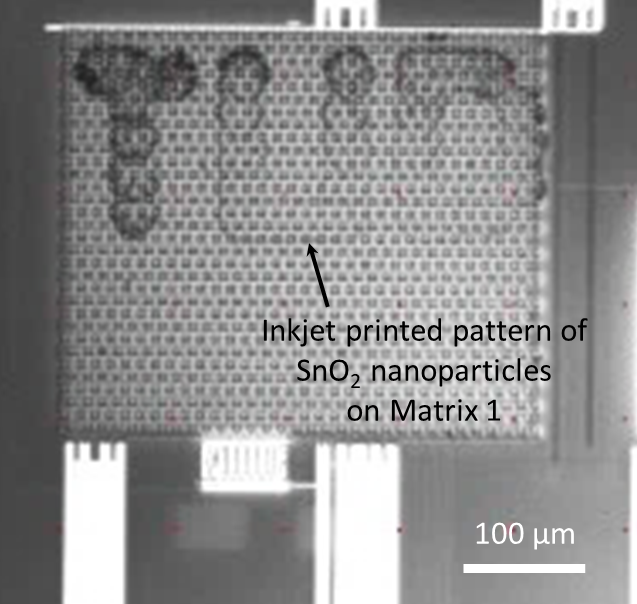
Figure S25.** Optical microscopy image of the letters “TUD” printed on Matrix 1 using SnO_2_ NP ink **
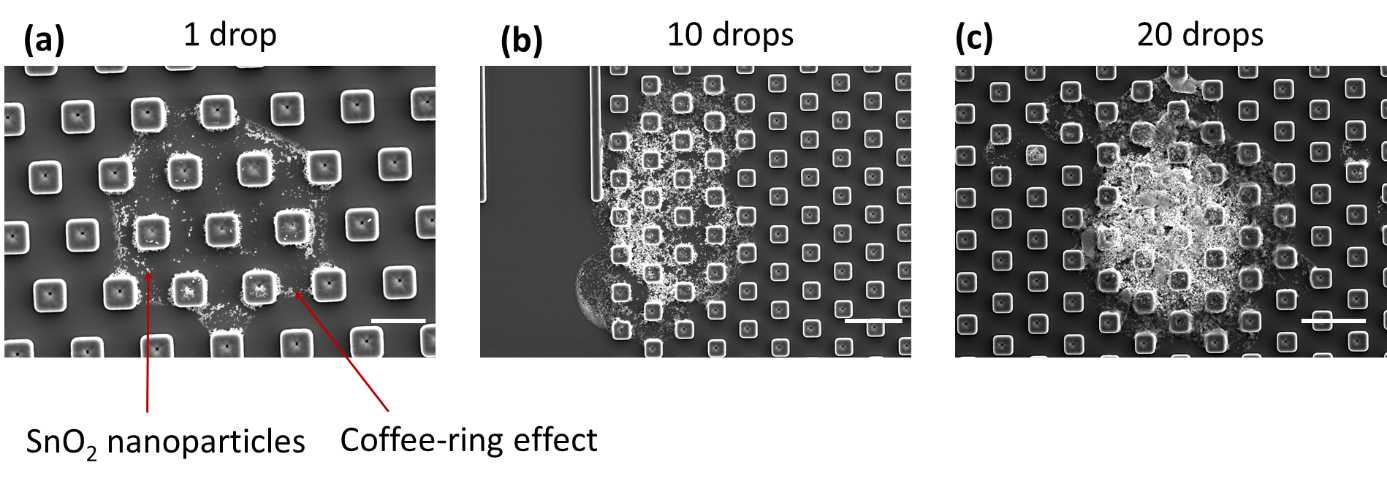
**(10 pL droplets).

**Figure S26.** FESEM images of SnO_2_ NP ink droplets printed on the sensor electrodes with (a) 1, (b) 10, and (c) 20 droplets deposited at the same spot. The volume of a single droplet was 10 pL. Higher density of the NPs is observed at the periphery of the printed-spot (see Figure a), due to coffee ring effect. Scale bars in a represent 10 µm, and in b, c represent 20 µm.

**
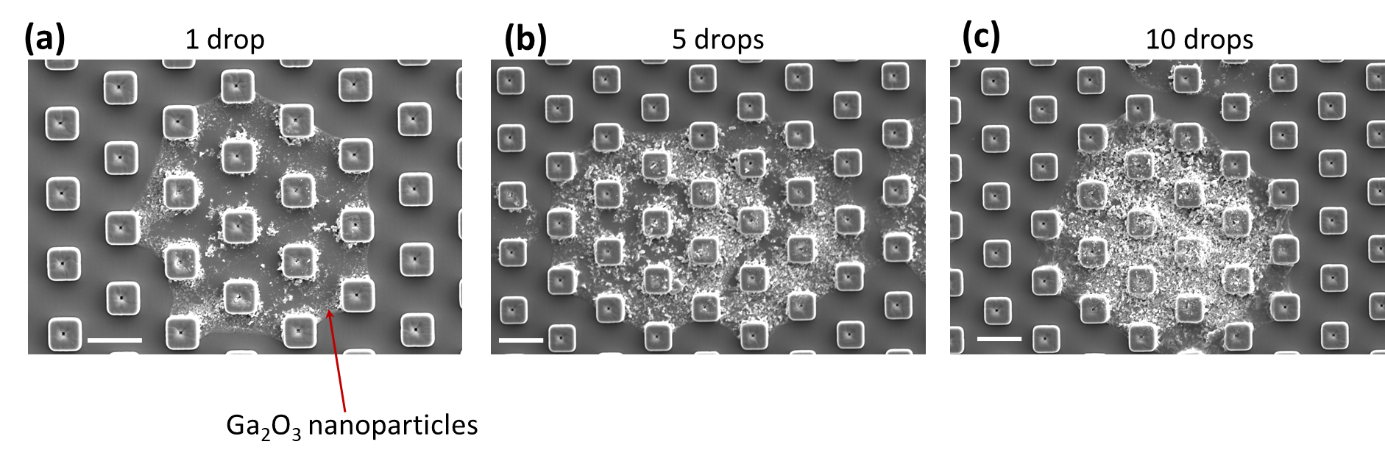
**
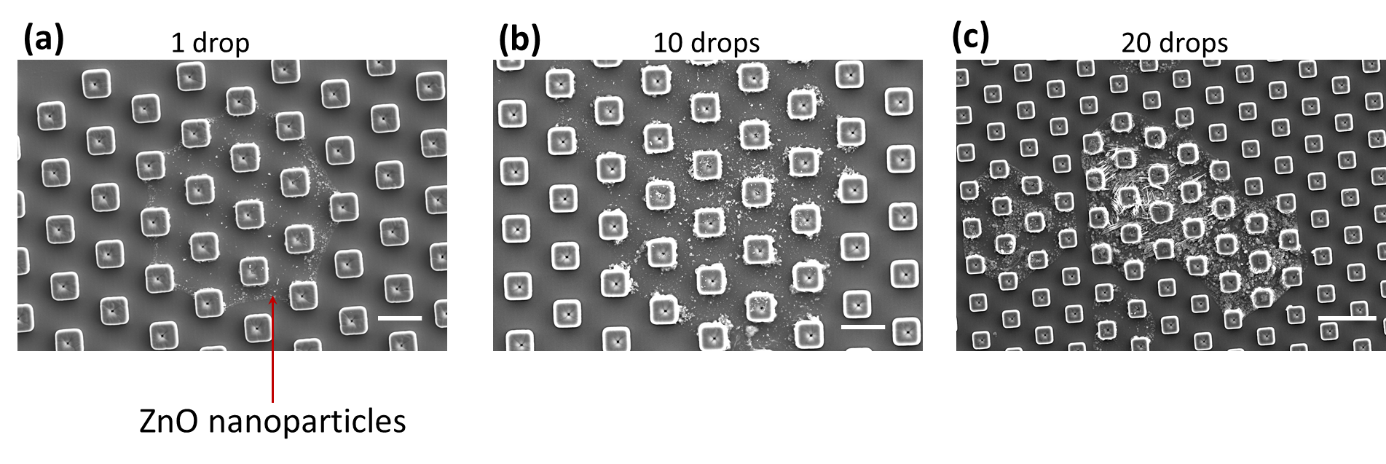
**Figure S27.** FESEM images of ZnO NP ink droplets printed on the sensor electrodes with (a) 1, (b) 10, and (c) 20 droplets deposited at the same spot. The volume of a single droplet was 10 pL. Scale bars in a, b represent 10 µm, and in c represent 20 µm.

**Figure S28.** FESEM images of Ga_2_O_3_ NP ink droplets printed on the sensor electrodes with (a) 1, (b) 5, and (c) 10 droplets deposited at the same spot. The volume of a single droplet was 10 pL. Scale bars in a, b and c represent 10 µm.


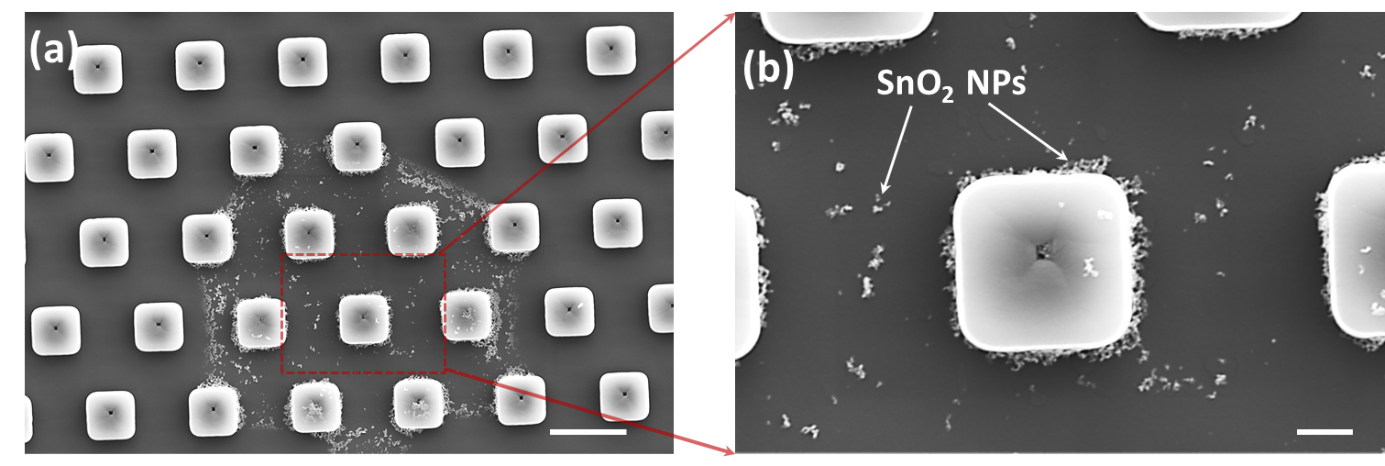


**Figure S29**. (a) FESEM image of SnO_2_ NP ink droplet (1 droplet, 10 pL) printed on the sensor electrode. (b) Magnified view of the red-dashed region in a. Scale bars in a represent 10 µm, and in b represent 2 µm.

**
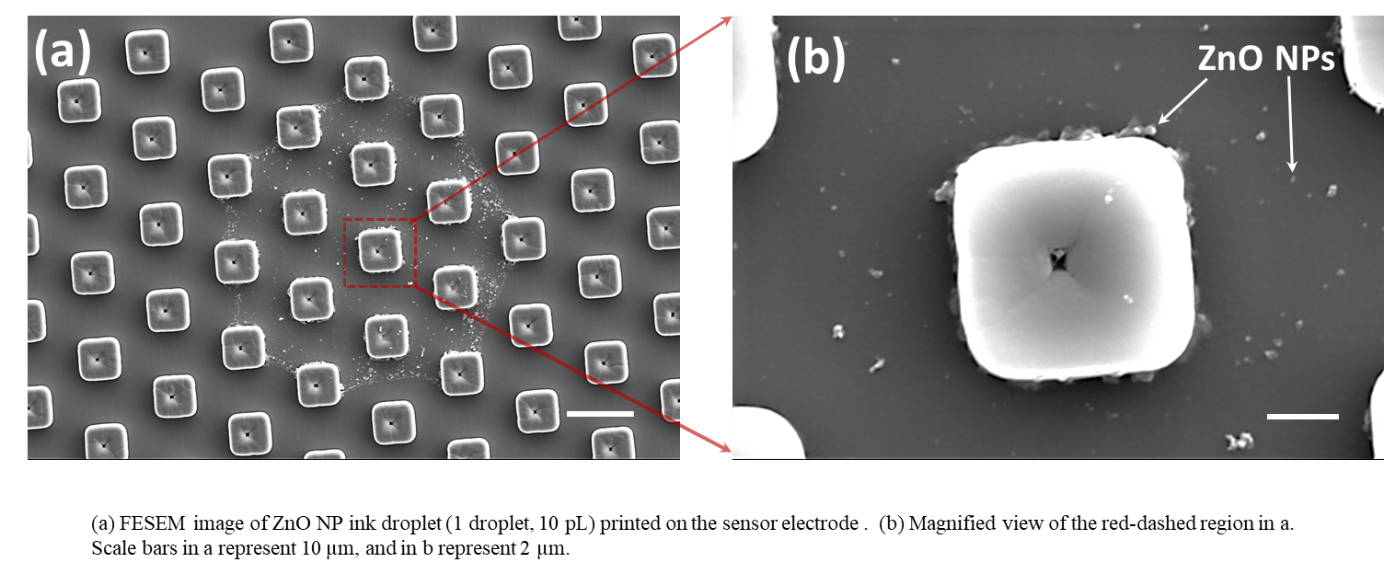
**

**Figure S30**. (a) FESEM image of ZnO NP ink droplet (1 droplet, 10 pL) printed on the sensor electrode. (b) Magnified view of the red-dashed region in a. Scale bars in a represent 10 µm, and in b represent 2 µm.

**
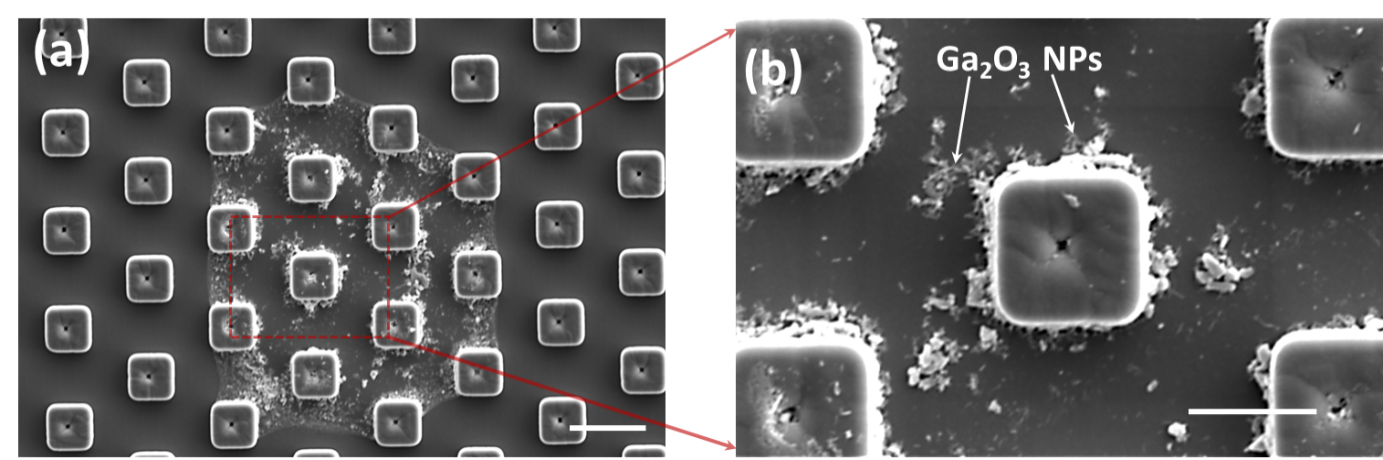
**

**Figure S31**. (a) FESEM image of Ga_2_O_3_ NP ink droplet (1 droplet, 10 pL) printed on the sensor electrode. (b) Magnified view of the red-dashed region in a. Scale bars in a represent 10 µm, and in b represent 2 µm.


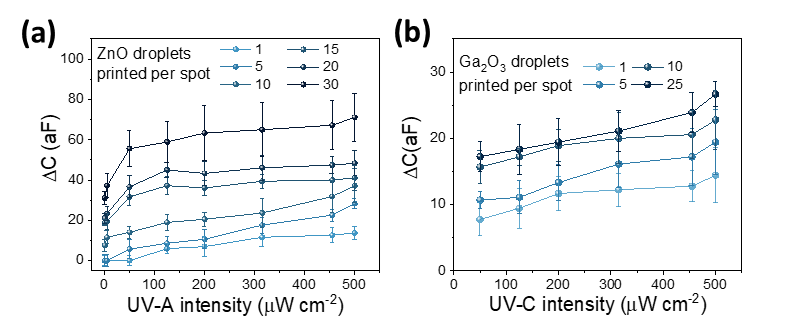


**Figure S32.** Capacitance variation of the sensor pixels inkjet-coated with different numbers of (a) ZnO and (b) Ga_2_O_3_ ink droplets under varying intensities (0.5 – 500 µW cm^-2^) of UV-A and UV-C, respectively.


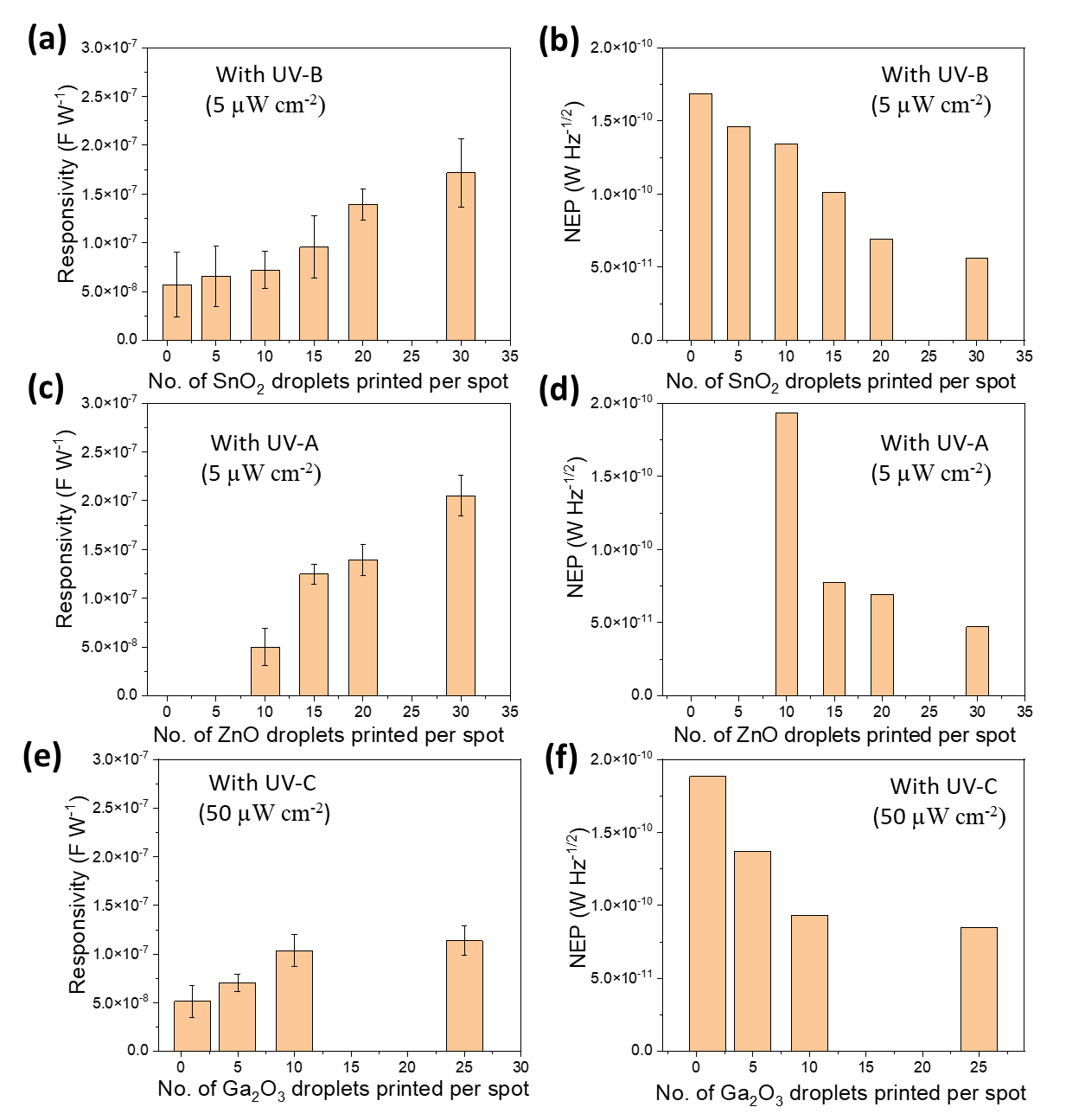


**Figure S33.** Responsivity and corresponding NEP values of the sensor pixels inkjet-coated with different numbers of (a,b) SnO_2_ (c,d) ZnO and (e,f) Ga_2_O_3_ NP ink droplets under constant UV intensities (indicated inside each figure).

**
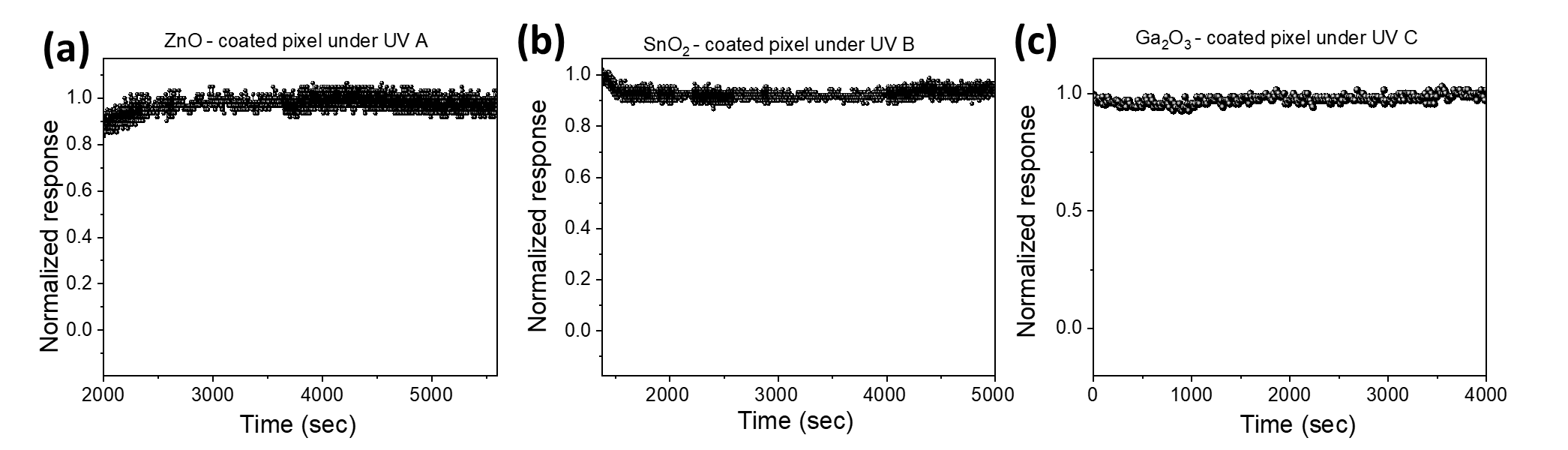
Figure S34**. The normalized responses of (a) ZnO, (b) SnO_2_ and (c) Ga_2_O_3_ coated pixels under long (1 hour) exposure of UV A, UV B and UV C, respectively.


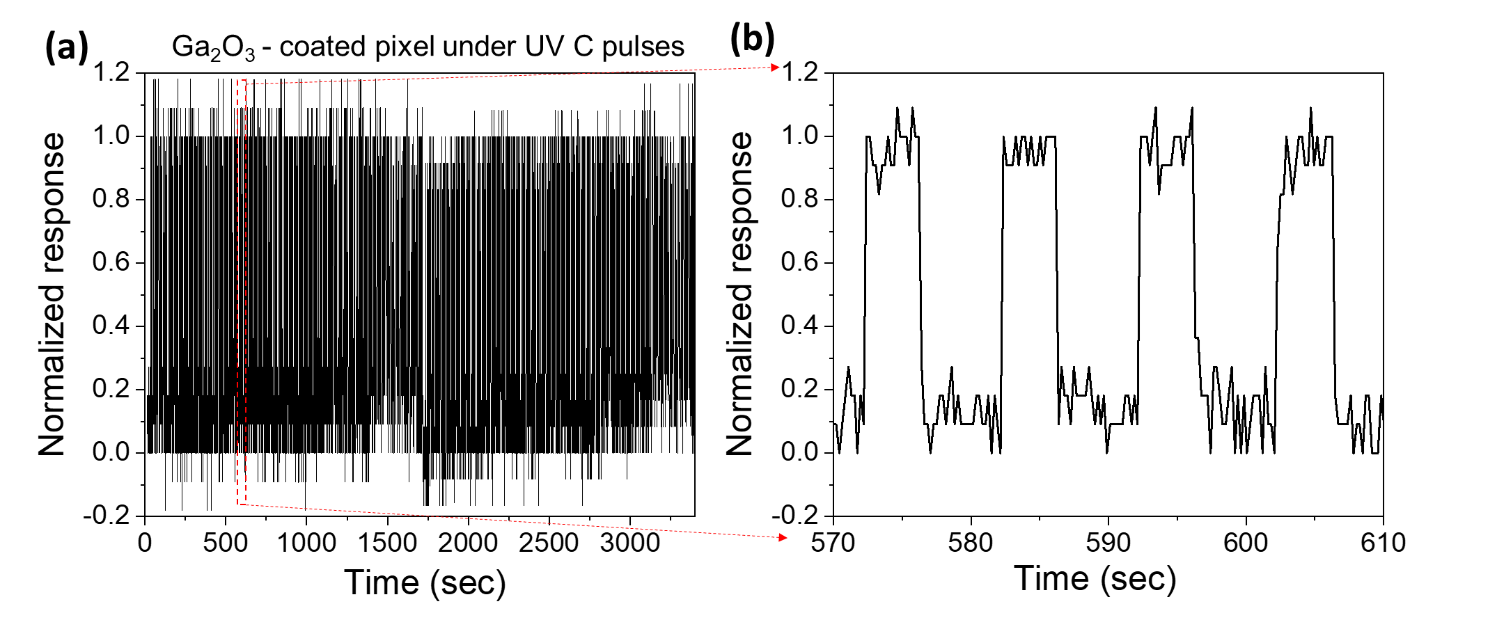
To evaluate device stability, we performed measurements under continuous UV illumination for 1 hour on MOX nanoparticle-coated chips (see Fig. S34). We observed no large drift in the capacitance response during this extended exposure, indicating stable device operation under prolonged illumination conditions.

**Figure S35**. (a) Normalized response of Ga_2_O_3_ coated pixel under repeated UV C pulses (4s on, 6s off, 340 cycles). (b) Zoomed in view of four UV C on-off cycles.

To demonstrate cycling stability under repeated UV illumination cycles, we have monitored the Ga_2_O_3_ coated pixels and tested them under repeated UV-C exposure cycles (340 cycles). The device exhibited consistent and reproducible responses (Fig. S35), further supporting the robustness of the devices.

**
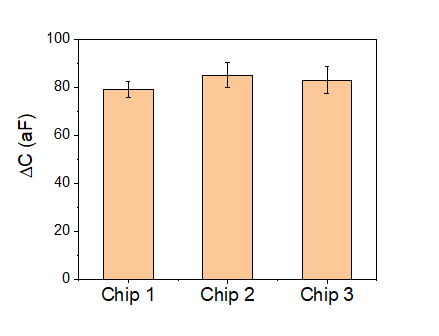
Figure S36**. Response from the inkjet-coated with SnO_2_ NP droplets (1 droplet with a volume of 10 pL) on three different chips under UV-B exposure (500 µW cm^-2^).


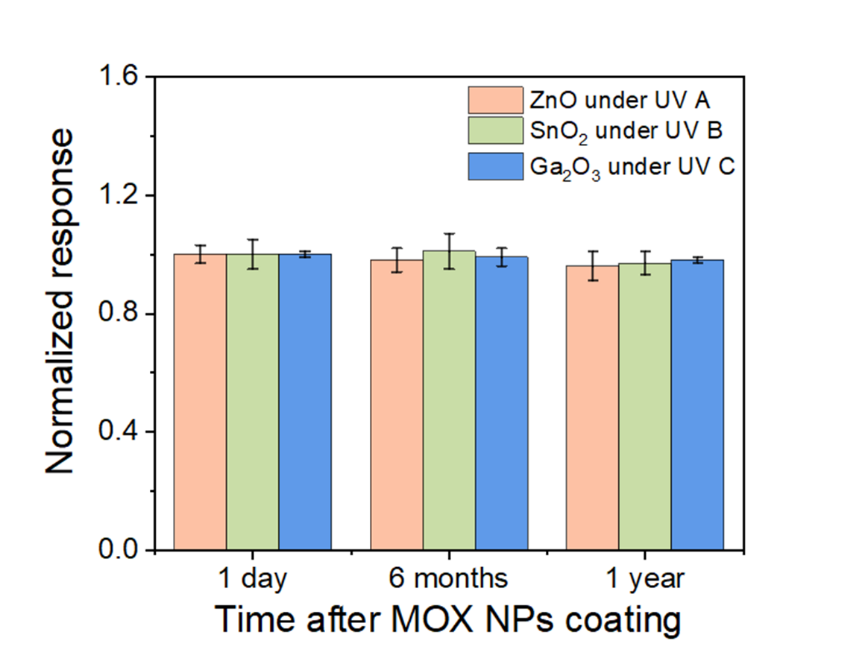
To demonstrate reproducibility, we compared the performance of three devices with SnO_2_ inkjet-printed (single droplet, 10 pL) on them and exposed under UV-B illumination with an intensity of 500 µW cm⁻² (see Fig. S36). The devices exhibit consistent capacitance responses within a similar range, indicating good device-to-device uniformity. These results confirm that the fabricated devices demonstrate reproducible performance under identical operating conditions.

**Figure S37.** Normalized responses of the ZnO, SnO_2_ and Ga_2_O_3_ coated pixels under UV A, UV B and UV C, respectively after 1 day, 6 months and 1 year of functionalization. The devices are stored in ambient.

To evaluate long-term stability, the MOX nanoparticle-coated devices were stored under ambient conditions, and their UV responses were measured after 1 day, 6 months, and 1 year following functionalization. We observed that the devices retained 96-98% of their original performance even after 1 year of storage (see Fig. S37), demonstrating excellent long-term stability.

**
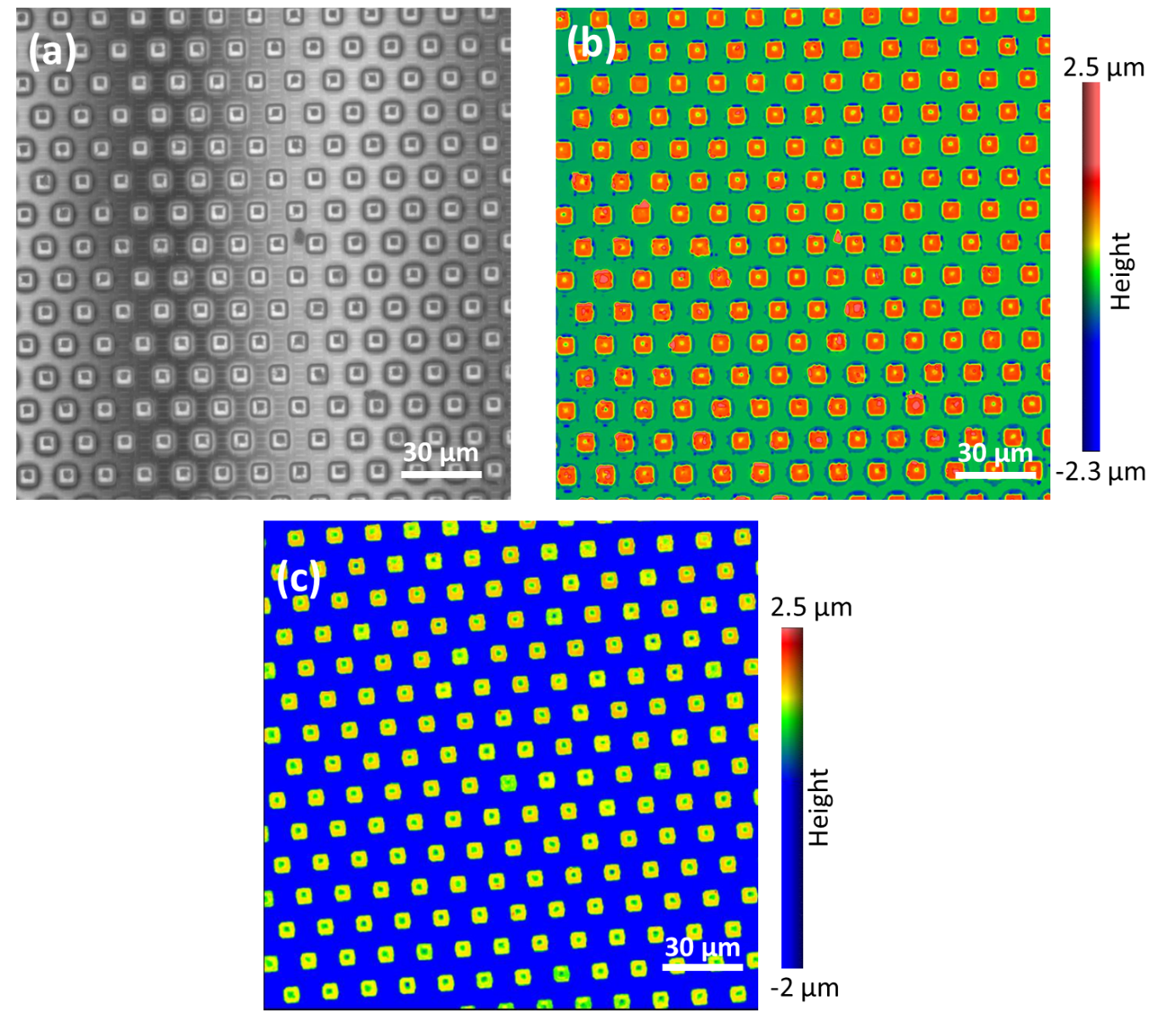
Figure S38.** (a) Optical microscopy image of Matrix 1 of SnO_2_-NPs spin-coated film. (b) The optical profilometry of the SnO_2_-NPs spin-coated film. The root mean square surface roughness of the area shown in b is ~ 0.63 µm. (c) The optical profilometry of the matrix 1 of an empty chip. The root mean square surface roughness of the area shown in c is ~ 0.58 µm. This indicates that the nanoparticle coating does not introduce significant additional surface irregularities.


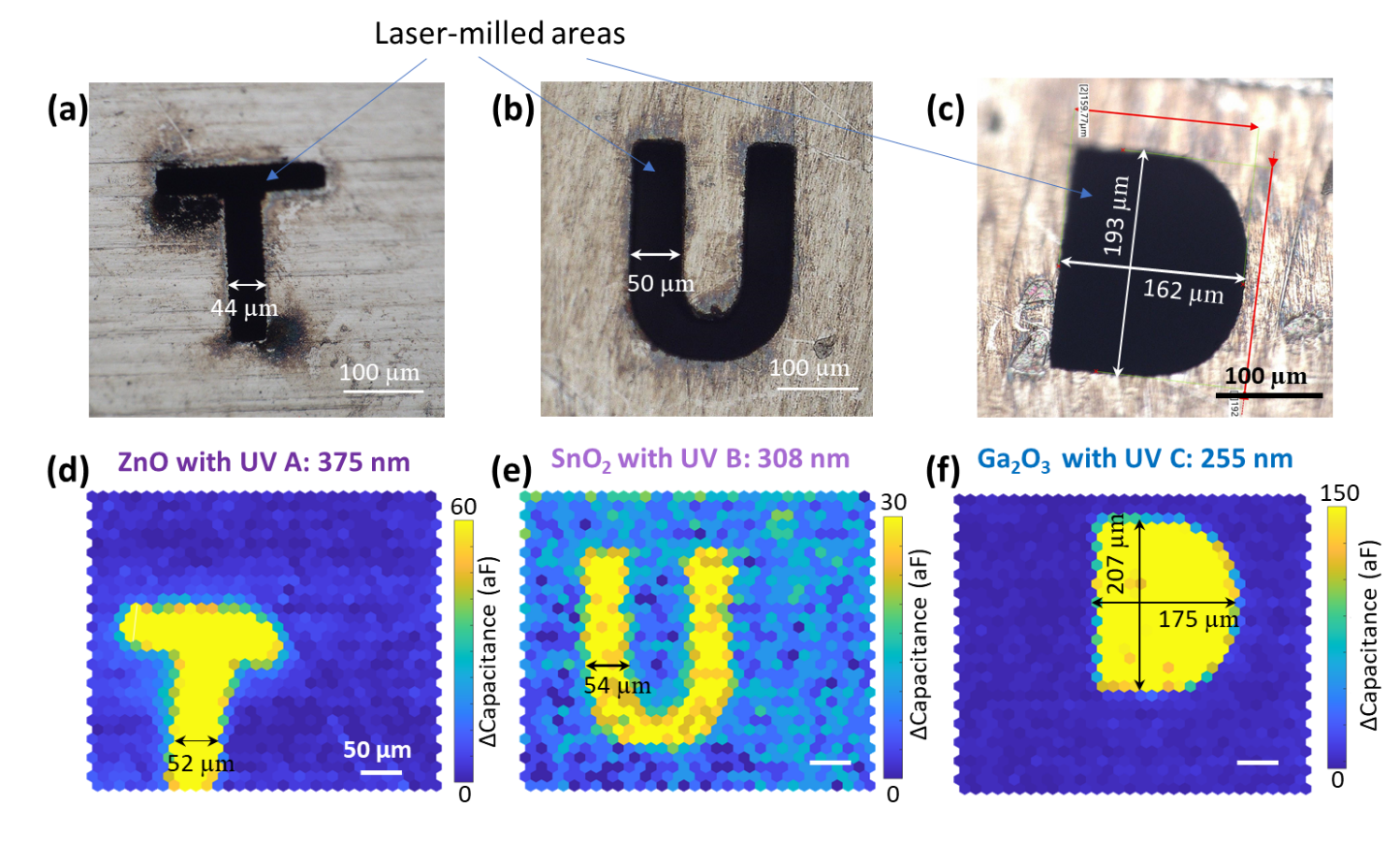

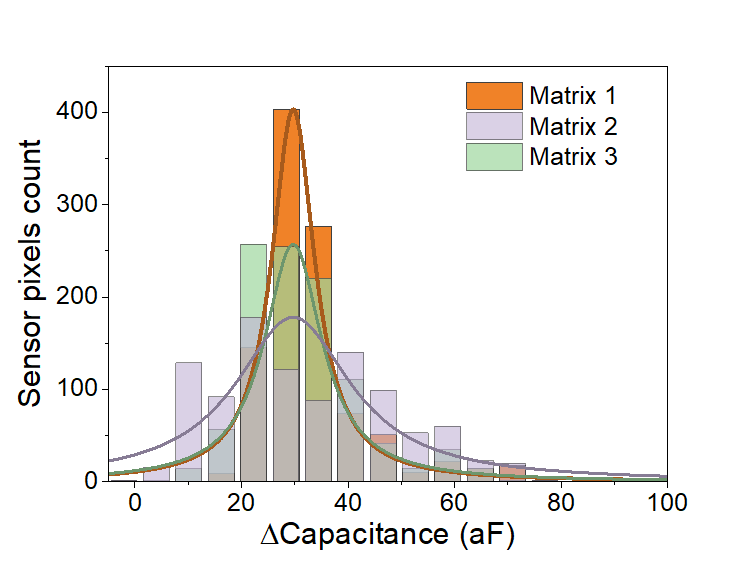
**Figure S39.** The distribution of capacitive responses at the Matrix 1, 2 and 3 of the SnO_2_ NP spin-coated chip under the UV-B exposure of intensity 250 µW cm^-2^.

**Figure S40.** (a-c) Optical microscope images of the steel optical masks containing laser-milled patterns in the shapes of the letters “T”, “U”, and “D”. (d-f) Corresponding responses of ZnO, SnO_2_, and Ga_2_O_3_ coated pixels under UV-A (375 nm), UV-B (308 nm), and UV-C (255 nm) illumination, respectively. For comparison, the dimensions of the laser-milled patterns on the steel masks and the resulting projected patterns on the PCS array are shown inside the figures. Scale bars in e, f represent 50 µm.

**Note 4. Dynamic tracking of a moving UV light source**


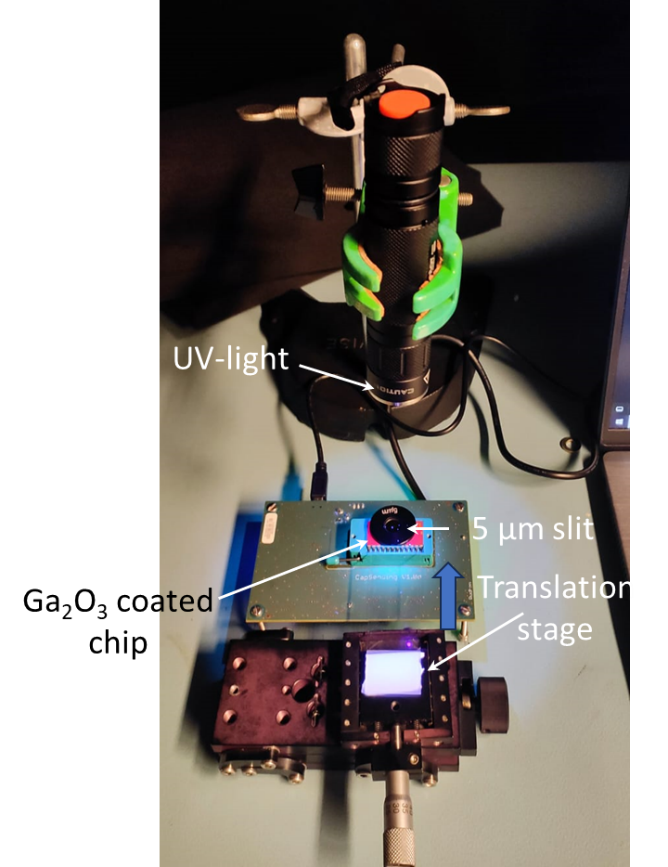
For this experiment, the Ga_2_O_3_ coated chip was employed and it was illuminated with UV-C through a 5 µm slit positioned directly on top of the chip (Figure S41). The slit position was manually adjusted using a micro-translation stage, while the corresponding images were recorded by the PCS array. A bright line of increased capacitance consistently appeared in the array, corresponding to the central maximum of the diffraction pattern generated by the slit. As the slit was translated along the length of Matrix 1, this bright line shifted accordingly, and the corresponding responses were captured, as shown in Figure S42.


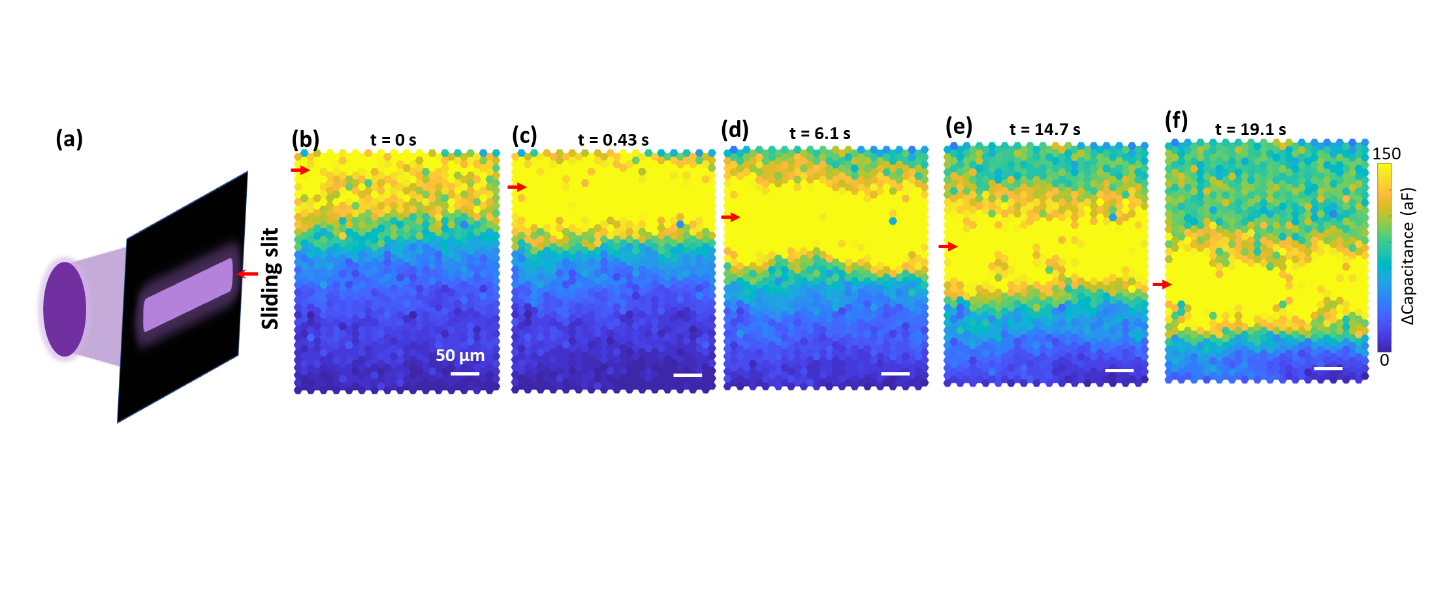
**Figure S41.** Image of the experimental setup, showing a 5 µm-wide slit positioned on top of the chip. The slit was translated across the PCS arrays using a manual translation stage, while a 255 nm UV light illuminated the Ga_2_O_3_ coated chip. The direction of slit movement is indicated by the blue arrow.

**Figure S42.** (a) Schematics of UV light exposed from a sliding slit to allow UV-C radiation to pass through and project on the Ga_2_O_3_ coated chip. (b-f) Corresponding responses from the Ga_2_O_3_ coated chip at different times while translating the slit across the whole length of Matrix 1. Scale bars in b-f represent 50 µm.
